# Progressive Visceral Leishmaniasis Is Driven by Dominant Parasite-induced STAT6 Activation and STAT6-dependent Host Arginase 1 Expression

**DOI:** 10.1371/journal.ppat.1002417

**Published:** 2012-01-19

**Authors:** E. Yaneth Osorio, Weiguo Zhao, Claudia Espitia, Omar Saldarriaga, Leo Hawel, Craig V. Byus, Bruno L. Travi, Peter C. Melby

**Affiliations:** 1 Research Service, Department of Veterans Affairs Medical Center, South Texas Veterans Health Care System, San Antonio, Texas, United States of America; 2 Department of Medicine, The University of Texas Health Science Center, San Antonio, Texas, United States of America; 3 Department of Microbiology and Immunology, The University of Texas Health Science Center, San Antonio, Texas, United States of America; 4 Division of Biomedical Sciences, University of California, Riverside, California, United States of America; McGill University, Canada

## Abstract

The clinicopathological features of the hamster model of visceral leishmaniasis (VL) closely mimic active human disease. Studies in humans and hamsters indicate that the inability to control parasite replication in VL could be related to ineffective classical macrophage activation. Therefore, we hypothesized that the pathogenesis of VL might be driven by a program of alternative macrophage activation. Indeed, the infected hamster spleen showed low NOS2 but high arg1 enzyme activity and protein and mRNA expression (p<0.001) and increased polyamine synthesis (p<0.05). Increased arginase activity was also evident in macrophages isolated from the spleens of infected hamsters (p<0.05), and arg1 expression was induced by *L. donovani* in primary hamster peritoneal macrophages (p<0.001) and fibroblasts (p<0.01), and in a hamster fibroblast cell line (p<0.05), without synthesis of endogenous IL-4 or IL-13 or exposure to exogenous cytokines. miRNAi-mediated selective knockdown of hamster arginase 1 (arg1) in BHK cells led to increased generation of nitric oxide and reduced parasite burden (p<0.005). Since many of the genes involved in alternative macrophage activation are regulated by Signal Transducer and Activator of Transcription-6 (STAT6), and because the parasite-induced expression of arg1 occurred in the absence of exogenous IL-4, we considered the possibility that *L. donovani* was directly activating STAT6. Indeed, exposure of hamster fibroblasts or macrophages to *L. donovani* resulted in dose-dependent STAT6 activation, even without the addition of exogenous cytokines. Knockdown of hamster STAT6 in BHK cells with miRNAi resulted in reduced arg1 mRNA expression and enhanced control of parasite replication (p<0.0001). Collectively these data indicate that *L. donovani* infection induces macrophage STAT6 activation and STAT6-dependent arg1 expression, which do not require but are amplified by type 2 cytokines, and which contribute to impaired control of infection.

## Author Summary

Visceral leishmaniasis (VL), caused by the intracellular protozoan *Leishmania donovani*, is a progressive, potentially fatal infection found in many resource-poor regions of the world. We initiated these studies of an experimental model of VL to better understand the molecular and cellular determinants underlying this disease. We found that host macrophages or fibroblasts, when infected with *Leishmania donovani* or exposed to products secreted by the parasite, are permissive to infection because they fail to metabolize arginine to generate nitric oxide, the effector molecule needed to kill the intracellular parasites. Instead, the infected host cells are activated in a way that leads to the expression of arginase, an enzyme that metabolizes arginine to produce polyamines, which support parasite growth. This detrimental activation pathway was dependent on the parasite-induced activation of the transcription factor STAT6, but contrary to the previously accepted paradigm, did not require (but was amplified by) the presence of polarized Th2 cells or type 2 cytokines. Knockdown of host arginase or STAT6 enhanced control of the infection, indicating that this activation pathway has a critical role in the pathogenesis of the disease. Interventions designed to inhibit the STAT6-arginase-polyamine pathway could help in the treatment or prevention of VL.

## Introduction

In humans, active visceral leishmaniasis (VL), caused by the intracellular protozoan *Leishmania donovani*, is a progressive, potentially fatal infection characterized by chronic, fever, hepatosplenomegaly, pancytopenia, and profound cachexia. VL remains a significant cause of morbidity and mortality in the developing world; hundreds of thousands of people have died in recent years in epidemics in Sudan and India.

Most experimental studies of infection with the visceralizing *Leishmania* (*L. donovani* and *L. infantum/chagasi*) have used the murine infection model. Mice are genetically resistant or susceptible to *L. donovani*, but even susceptible strains are able to contain the infection without overt disease [1]. *L. donovani*-infected mice mount a vigorous anti-leishmanial type 1 CD4+ and CD8+ T cell response that leads to control of the infection, primarily through the upregulation of inducible nitric oxide synthase 2 (iNOS or NOS2) and generation of nitric oxide (NO) in the spleen and liver [2]–[4]. Susceptibility in the murine *L. donovani* infection model is related to the expression of IL-10 [5], [6] and TGF-β [7]. Notably, IL-4, which has a prominent role in the immunopathogenesis of murine *L. major* infection (reviewed in [8]), appears to have a limited role in the pathogenesis of murine *L. donovani* infection [9]. While study of the chronic, self-controlled infection in mice has been instrumental in dissecting mechanisms of immunity and susceptibility, this model may be limited in representing the mechanistic underpinnings of progressive visceral disease.

The underlying immunopathogenic mechanisms related to human VL have not been fully elucidated. In human VL there is elevated expression of type 1 cytokines (IFN-γ and IL-12) in the plasma [10], [11] and in the infected lymph node, bone marrow, and spleen [12]–[14]. Paradoxically, this robust type 1 cytokine response, which typically mediates control of intracellular pathogens, does not mitigate the relentlessly progressive disease in humans. Several cytokines known to impair macrophage-mediated killing of *Leishmania* [15] have been postulated to have a detrimental role in human VL [16], [17]. The Th2 cytokines, interleukin (IL)-4 and IL-13, which play a prominent role in promotion of disease in some experimental models of *Leishmania* infection [8], were found to be increased in the serum of patients with active VL in some [18]–[21], but not all studies [22]–[24]. The importance of IL-10 in the pathogenesis of human VL is more clearly established [16]. Patients with VL have elevated levels of IL-10 in serum or plasma [22]–[25] and increased IL-10 mRNA expression in the spleen and bone marrow [13], [14], [19]. In vitro neutralization of IL-10 in peripheral blood mononuclear cell cultures from patients with VL resulted in enhancement of type 1 T cell responses to *Leishmania* antigens [12], [26], and neutralization of IL-10 in splenic aspirates promoted parasite clearance [27]. Impairment of signaling pathways in human macrophages infected in vitro with *Leishmania* is also well described (reviewed in [28]) and may play a role in human VL by rendering the infected cells less responsive to activating stimuli.

In light of the ineffective killing of *L. donovani* in human VL, it is pertinent to consider the activation phenotypes of macrophages. Classically activated macrophages are primed by proinflammatory cytokines, most notably IFN-γ, and triggered by microbial products to produce antimicrobial mediators such as NO and reactive oxygen species. These macrophages play a critical role in the protection against intracellular pathogens such as *Leishmania* [2], [29]–[35]. Macrophages exposed to type 2 cytokines (IL-4 and IL-13) were thought initially to be in a deactivated state because of blunting of the pro-inflammatory cytokine response, oxidative burst, or NO response [15]. However, it is now recognized that these macrophages are not paralyzed, but in fact display a different activation program. Alternatively activated macrophages (AAMs), as introduced by Gordon and colleagues in describing the phenotype of macrophages activated in the presence of IL-4 (and later IL-13) [36], [37], fail to produce NO, have pronounced arginase activity (which competes with NOS2 for the common substrate arginine), and fail to control the intracellular replication of pathogens, including *Leishmania* [38], [39]. Recently, a role for IL-21 in the amplification of arginase-producing alternatively activated macrophages has been identified [40]. Alternatively activated macrophages play an important role in dampening tissue inflammation, and mediating tissue repair and wound healing.

AAMs play a role in the pathogenesis of protozoal infections [41], and several lines of evidence from the murine *L. major* infection model identified an important role of AAMs in promoting *Leishmania* infection. First, the constitutive expression of arg1 was higher in macrophages from *L. major*-susceptible compared to resistant mice [42]. Second, arg1 induction correlated with lesion size in mice infected with *L. major* [43], [44]. Third, AAMs failed to control the intracellular replication of pathogens, including *Leishmania* [38], [39]. Fourth, the upregulation of arginase by IL-4, IL-10, and TGF-β was associated with impaired capacity to kill intracellular *L. major* [42]–[44]. Finally, inhibition of arginase decreased disease and parasite burden in *L. major* infected mice and macrophages [43], [44]. The increased expression of host arg1 has two important downstream effects that can promote *Leishmania* infection: (1) arginase competes with NOS2 for the common substrate, arginine, thereby reducing the generation of the antimicrobial molecule NO; and (2) arginase activity leads to the generation of polyamines that can be scavenged through uptake receptors [45] to promote *Leishmania* growth [46].

Unfortunately, there remain significant deficits in our understanding of the molecular and cellular determinants underlying VL pathogenesis. The Syrian hamster (*Mesocricetus auratus*) affords a unique opportunity to address questions related to the pathogenesis of visceral leishmaniasis, because the clinicopathological features of the hamster model of VL mimic active human disease. We, and others, demonstrated that despite progressive disease hamsters with VL mount a vigorous type 1 cellular immune response [47]–[49], an immunological event that is typically associated with disease control and resolution. This paradoxical finding was reminiscent of the findings in humans [13], [14], and suggested that the inability to control parasite replication could be related to ineffective IFN-γ-mediated induction of classical macrophage activation. Indeed, we found that the expression of NOS2 and production of NO, which is the primary mechanism by which mice control *Leishmania* infection [2], [29]–[35], was low during the progressive course of disease in hamster VL [48], [49]. Because of the low NOS2 expression in hamsters with VL we hypothesized that during progressive disease macrophages would default toward and/or be driven toward an alternatively activated phenotype. Indeed, the expression of arg1 and the production of polyamines was dominant in progressive VL. However, distinct from the prevailing paradigm of cytokine-mediated alternative activation of macrophages, we found that the *L. donovani*-induced activation of STAT6 and arg1 expression and polyamine production in macrophages and fibroblasts did not require the presence of a polarized Th2 response or synthesis of type 2 cytokines. The critical importance of the parasite-induced STAT6-arg1 pathway in infected cells was demonstrated by finding enhanced control of infection following either STAT6 or arg1 knockdown.

## Materials and Methods

### Ethics statement

This study was carried out in strict accordance with the recommendations in the Guide for the Care and Use of Laboratory Animals of the National Institutes of Health. The protocol was approved by the Institutional Animal Care and Use Committee of the University of Texas Health Science Center at San Antonio and the Institutional Animal Care and Use Committee of the South Texas Veterans Health Care System.

### Hamsters and mice

6-8 week old inbred Chester Beatty Syrian golden hamsters (*Mesocricetus auratus*) were obtained from our own established breeding colony at the South Texas Veterans Health Care System Veterinary Medical Unit. 6-week old BALB/c mice were obtained from Charles River Laboratories.

### Isolation of primary macrophages and fibroblasts

Splenic macrophages were isolated from a spleen cell suspension by adherence to plastic culture dishes. Resident peritoneal macrophages were isolated from mice or hamsters by peritoneal lavage with DMEM containing 2% heat-inactivated fetal calf serum (HIFCS) and cultured in complete DMEM (DMEM plus 2mM glutamine, 1 mM Sodium pyruvate (Gibco), 1X MEM aminoacids solution (Sigma), 50 µM β-mercaptoethanol, 10 mM Hepes, 100 U/ml penicillin, and 100 mg/ml streptomycin) with 2–10% HIFCS. After overnight incubation, non-adherent cells were removed and adherent cells cultured in complete DMEM with 0.4% bovine serum albumin (BSA) or in Opti-MEM (Invitrogen) plus 1% HIFCS for in vitro infections. Hamster primary fibroblasts were obtained following the protocol described to isolate mouse splenic primary fibroblasts [50]. In brief, spleens were harvested from uninfected hamsters, treated with collagenase D to obtain a single cell suspension, and cultured in RPMI 1640 containing 10% of heat inactivated FBS, 10 mM Hepes, 1 mM sodium pyruvate (1 mm), 1X MEM aminoacids solution (Sigma), 100 IU/mL penicillin, 100 mg/mL streptomycin (Cellgro) and 50 mM 2-mercaptoethanol. The non-adherent cells were removed at 24, 48 and 72 h of culture and the adherent cells were cultured to reach confluence over 2 weeks. The fibroblast-like monolayer was detached with Trypsin/EDTA and cultured overnight in DMEM plus 2% FBS prior infection experiments. The purity of the fibroblast population was determined to be >80% using PE labeled-antibody against the ER-TR7 antigen (Santa Cruz) and flow cytometry.

### Parasites and infection

*Leishmania donovani* (MHOM/SD/001S-2D) promastigotes were cultured as described previously [51]. Hamsters were infected by intracardial injection and mice by intravenous injection of 10^6^ peanut agglutinin purified metacyclic promastigotes [51] of a *L. donovani* strain transfected with an episomal vector containing the luciferase (luc) reporter gene [52]. The parasite burden was measured in 100 mg of tissue homogenized in PBS and the luminescent counts were transformed to number of parasites by interpolation from a standard curve of luciferase activity and number of amastigotes. For in vitro infections, stationary phase promastigotes obtained from 5–6 day old cultures were either non-opsonized, or opsonized with fresh, complement-containing normal mouse or hamster serum (20% in DMEM), or freeze-thawed hamster serum obtained from 6-week infected hamsters (contained high titer of anti-*Leishmania* antibody) for 30 min at 37°C and 5% CO_2_, washed with PBS and used immediately to infect the adherent primary macrophages or splenic fibroblasts, or serum-starved (2% HIFCS) BHK fibroblast cells. Cells were infected at a promastigote to host cell ratio of 10∶1. Four hours after the infection extracellular parasites were removed by repeated (at least 3 times) washing with PBS and the infected cells cultured thereafter in the low-serum medium described above. For parasite burden determinations, the complete removal of extracellular parasites was verified by microscopic inspection. The level of infection after initial phagocytosis was assessed 4 hrs after infection in Giemsa-stained preparations enumerated by microscopy. Typically at 4 hrs post-infection there were 1–2 parasites per infected BHK cell (range 1 to 7) and >75% of cells were infected. In experiments where parasite killing was assessed an equivalent rate of initial (4 hr) infection was confirmed and the parasite burden determined 4–72 hrs after infection by luminometry.

### Measurement of NOS2 and arginase enzymatic activity and arginase protein expression

NOS2 activity (NO production) was estimated by the measurement (Griess assay) of nitrites and nitrates in supernatants of unstimulated cells or cells stimulated with IFN-γ (10% v/v of hamster recombinant IFN-γ supernatants) plus 1 µg/mL lipopolysaccharide (LPS; *E. coli* serotype 0111:B4; Sigma) as described previously [48], [49]. The enzymatic activity of arginase was determined in 100,000 adherent splenocytes [53], peritoneal macrophages, or BHK fibroblasts cultured for 24–48 hrs in complete DMEM supplemented with 0.4% BSA by measuring the rate of urea formation from L-arginine in the presence of 1-phenyl-1,2-propanedione-2-oxime (ISPF) [54], [55]. Cells were stimulated with IL-4 (10% v/v) and/or LPS (1 µg/mL), or infected with *L. donovani* promastigotes or amastigotes. The measurement of arginase activity in tissue samples homogenized in PBS (5 mg/mL) was accomplished using the same method. Arg1 and GAPDH protein expression were determined by western blot after separation of 2 µg protein/lane from spleen homogenates and probing with 1 µg/mL goat anti-hamster arg1 polyclonal antibody or mouse anti-glyceraldehyde-3-phosphate dehydrogensase (Clone 6C5, Millipore) diluted 1∶500 in 3% nonfat milk in TBS-T + 0.5M NaCl and incubated at 4°C for 2 hours. After washing the membrane the primary antibody was detected with HPRT-conjugated rabbit anti-goat antibody diluted 1∶20,000 in 5% BSA in TBS-T for 1.5 hrs at room temperature. The anti-hamster arg1 antibody was produced by Genemed Synthesis, Inc. (San Antonio, TX) by immunizing goats with peptides (CFGTAREGNHKPGVDYLNNPPK and CGLVEKLKETVYDVKDY) derived from the hamster arg1 deduced amino acid sequence. The antibody did not react with *L. donovani* parasite lysates.

### Determination of tissue and cellular polyamine content

Polyamines (putrescine, cadaverine, spermine, spermidine, N-acetylspermine, and N-acetylspermidine) were quantified in acid-extracted uninfected and infected hamster spleen, or uninfected and in vitro infected macrophages using High Performance Liquid Chromatography and fluorescence-based detection as described [56]–[58].

### Sequences of hamster and *L. donovani* cDNAs

The cDNAs of the hamster arg1 (GenBank Accession number HM801029), arg2 (GenBank Accession number HM801027), STAT6 (GenBank Accession number HM801028), and *Leishmania donovani* arginase (GenBank Accession number DQ649412) were cloned and sequenced as we have described previously [49], [59].

### Determination of gene expression

The expression of hamster and mouse mRNAs and *L. donovani* arginase were determined in uninfected or infected tissue or cells by real time RT-PCR. In brief, the extracted RNA (RNeasy, Quiagen) was treated with DNAse (Turbo DNAse, Ambion), adjusted to 40–100 ng/µL and reverse transcribed in a final volume of 20 µL (High capacity reverse transcription kit, Applied Biosystems). 2–40 ng of reverse transcribed RNA was amplified with 400 nM of primers and 200 nM of Taqman probe in 15 µL of master mix (TaqMan, Universal PCR Master Mix, Applied Biosystem). The sequences (5′ to 3′) of the primers and probes (5′ 6-FAM and 3′ TAMRA Quencher) used to detect the specific hamster or mouse cDNAs are as follows: hamster and mouse arg1, forward, ACCTATGTGTCATTTGGGTGGA, reverse, GCAGATATGCAGGGAGTCACC, probe, TGCATGGGCAACCTGTGTCCTTTCT; hamster and mouse arg2, forward, AGCCTGGCAATAGGTACCATTA, reverse, TTCCAGATACAGTGGTGAGAGGT, probe, CCGGCACCGCCCAGATCTC; Hamster NOS2, forward, TGAGCCACTGAGTTCTCCTAAGG, reverse, TCCTATTTCAACTCCAAGATGTTCTG, probe, CGTGGACACTTCCTTTGTCTG TGCTCC; Mouse NOS2, forward, CCCAACAATACAAGATGACCCTAA, reverse, TCCAGGGATTCTGGAACATTCT, probe, ACCAAAATGGCTCCCCGCAGC; hamster STAT6, forward, GAAGCACCACTTTGCAACACA, reverse, GGCAGGTGACGGAACTCTTCT, probe, AGCTGGTGGCCACCATCAGACAAATAC; *L. donovani* arginase, forward, CGCGGACATCAACACTATGTCT, reverse, AAAGCACTCGGGAATGTTCTTG, probe, CTTGCACGGCTGCCCCTTATCGATC. The level of gene expression was determined by the comparative threshold method using uninfected BHK-21 cells (Syrian hamster fibroblast cell line) as a calibrator sample and the 18S ribosomal RNA (rRNA) gene (Applied Biosystems) as a reference (normalizer) gene. Hamster cytokine mRNA expression was determined as published [59].

### Generation of recombinant hamster IL-4 and IFN-γ

Hamster IL-4 was cloned [60] and inserted into the pMIB expression vector (Invitrogen) and a stably-transfected line was derived in insect SF9 cells with blastocidin selection. The bioactivity of the recombinant protein in blastocidin-free SF9-supernatants was confirmed using the STAT6-luciferase reporter assay (see below) and the supernatants were used at 10% v/v concentration. Recombinant hamster IFN-γ was generated and used to stimulate cells as described previously [48], [49].

### Measurement of STAT6 activation

Phospho-STAT6 was measured in resident peritoneal macrophages infected *in vitro* or in resident peritoneal macrophages obtained from hamsters with VL. In brief, after lysis of the red blood cells, macrophages were fixed with Phosflow fix buffer I (BD), permeabilized with Phosflow perm buffer III (BD), washed and blocked with 5% donkey serum, 2% BSA, and 0.05% sodium azide in PBS, and incubated overnight at 4°C with rabbit anti-human Phospho-STAT6 antibody (Tyr641, Cell Signaling) or isotype controls diluted 1∶100 in blocking buffer. After washing, the cells were stained for 1 hr at room temperature with the secondary antibody (Texas red labeled donkey anti-Rabbit antibody) and the mean fluorescence intensity and percentage of positive cells was determined by flow cytometry (FacsAria, BD). The fluorescence of isotype controls from infected and uninfected cells was used to determine the threshold fluorescense.

STAT6 phosporylation was also determined by immunoprecipitation and western blotting. In brief, spleens were homogenized in RIPA buffer with protease and phosphatase inhibitors (Santa Cruz), the supernatants cleared with Protein A/G agarose (Santa Cruz), and incubated at 4°C overnight with 1 µg of anti-STAT6 polyclonal antibody (M-20, Santa Cruz). The STAT6/antibody complex was then immunoprecipitated with Protein A/G agarose, the protein released from the beads by heating at 95°C, and resolved by SDS-PAGE and transferred to nitrocellulose membranes. Blots were blocked with 5% BSA in TBS-T and incubated overnight at 4°C with 1 µg/mL of anti-phospho STAT6 antibody (Cell Signaling). After washing, the primary antibody was detected with HRP-conjugated anti-rabbit secondary antibody (Cell Signaling) and substrate (West Pico, Thermo Scientific) followed by chemiluminescent detection (ECL; Amersham). GAPDH expression was determined as described above.

STAT6 activation was measured by a reporter assay using the BHK-21 cell line transfected with the luciferase reporter plasmid p(IE-IL4_RE_)_4_-LUC (a generous gift from Dr. Michael Berton, University of Texas Health Science Center, San Antonio, TX) that included 4 copies of a consensus STAT6 response element sequence. The p(IE-IL4RE)4-LUC reporter plasmid was initially used to determine the STAT6 domains required for IL-4-induced transcription [61] and has since been used extensively as a reporter for IL-4 induced STAT6 activation. The BHK-21 [IL-4_RE_]_4_ LUC cell line was regularly maintained and sub-cultured every 72 h in DMEM with 10% HIFCS and 2 µg/mL puromycin. When cells were to be infected they were cultured overnight in complete DMEM with 2% HIFCS without puromycin, seeded in 96-clear-bottom white plates (25,000/well), and infected at 10∶1 parasite:cell ratio using opsonized *L. donovani* promastigotes. After 4 hr of infection, excess extracellular parasites were removed and the infected cells cultured in complete DMEM with 2% HIFCS. The STAT-6 reporter activity was determined by luminometry using 20 µL of 1X lysis buffer and 80 µL of luciferin substrate from Promega.

### Knock down STAT-6 and arg1 by miRNAi

The expression of STAT6 and arg1 genes was knocked down using a vector-based approach. miRNAi inserts targeting the hamster STAT6 or arg1 sequences and containing the structural features of the pre-miRNA, were designed using the BLOCK-iT RNAi Designer program (Invitrogen) as follows: hamster arg1, top, TGCTGTGTATCAGCTGACTAT CATG TGTTTTGGCCACTGACTGACACATGATACAGCTGATACA, bottom, CCT GTGTATC AGCTGTATCATGTGTCAGTCAGTGGCCAAAA CACATGATAGTCA GCTGATACAC; hamster STAT6, top, TGCTGAACAGGATCTCCTTGTTGAA CGTTTTGGCCACTG ACTGACGTTCAACAGAGATCCTGTT, bottom, CCTG AACAGGATCTCTGTTGAA CGTCAGTCAGTGGCCAAAACGTTCAACAAGGAGATCCTGTTC. dsOligos were generated and cloned into pcDNA 6.2-GW/±EmGFP-miR according the manufacturer instruction (Block-it PolII miR RNAi Expression Vector kits, Version E, Invitrogen). The expression vector was purified with PureLink HiPure Plasmid DNA purification kit (Invitrogen) and transfected in low passage BHK-21 cells (obtained from ATCC; 150,000 cells per well/500 µL in 24 well plates) using 0.8 µg of plasmid and 1 µL of Lipofectamine 2000 in OptiMem (InVitrogen) as recommended in the instruction manuals. The pcDNA 6.2-GW/±EmGFP-miR-LacZ plasmid (Invitrogen), which expresses an irrelevant target sequence was used as a control in all the miRNAi experiments. The transfected cells were selected after 24 hr in DMEM with 10% HIFCS and 20 µg/ml of Blasticidin, and after 15 days of selection the stably transfected cells were cloned by limiting dilution and screened for the level of gene knockdown using real time RT-PCR. The selected cells were expanded and the efficiency of gene knockdown was determined at the mRNA level by real time RT-PCR and at the protein level by western blot. STAT6- and arg1-knockdown cells were transferred to 12-well plates (250,000 cells/1 mL DMEM with 2% HIFCS and 20 µg/ml of blasticidin) and infected for 4h with 1∶10 *L. donovani* opsonized promastigotes as above. The parasite burden was determined in equal numbers of cells at the specific time points by luminometry.

### Statistical analyses

Comparison between experimental groups was performed using one-way ANOVA. A parametric or non-parametric test was selected according the distribution of the raw data, followed by a post-test analysis for multiple groups as appropriate. All analyses were conducted using GraphPad InStat version 3.00 software for Windows 95 (GraphPad Software, San Diego California USA).

## Results

### Dominant expression of arg1 during progressive VL

**Figure 1:**
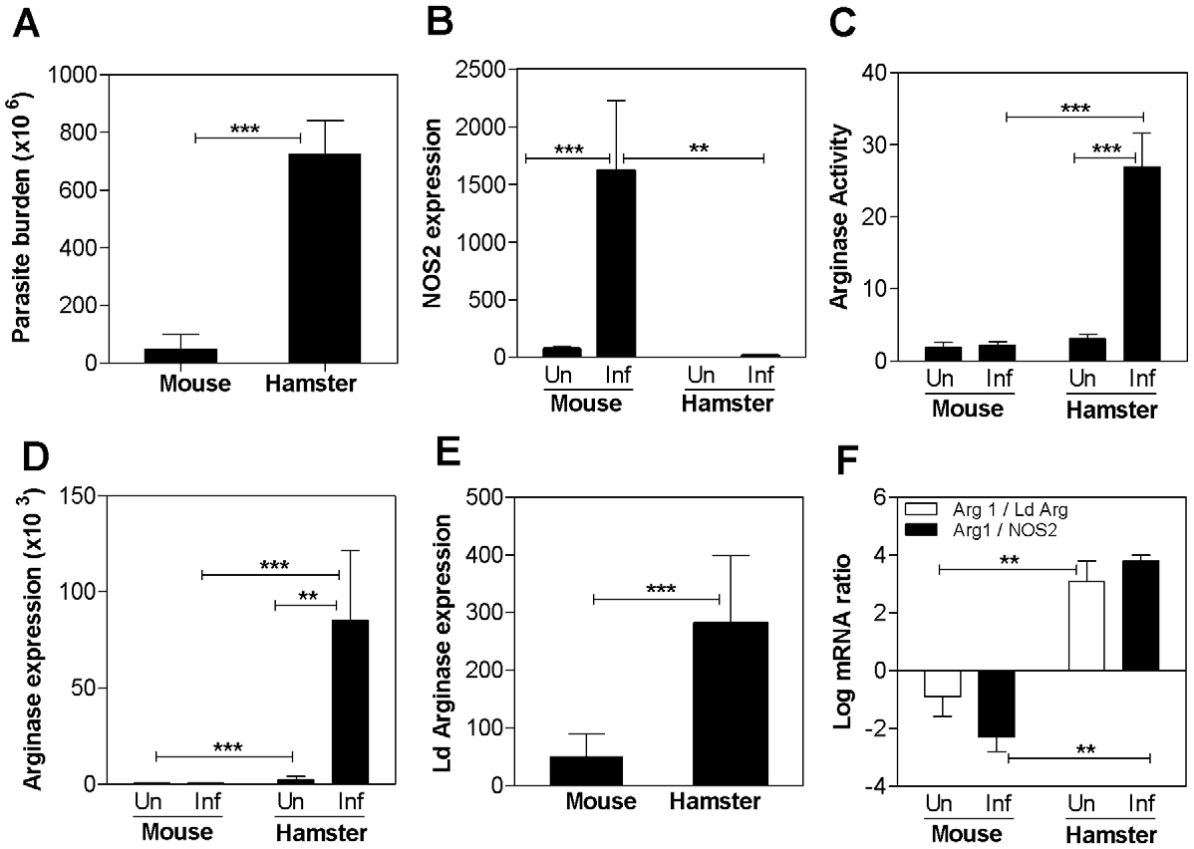
Dominant expression of arg1 in spleen tissue during progressive visceral leishmaniasis. **A**) Parasite burden in the spleens of hamsters and mice (n = 6 per group) infected with *L. donovani*-Luc for 6 weeks. The mean and standard deviation (SD) of the parasite burden, determined by luminometry and interpolation from a standard curve generated by enumeration and luciferase assay of amastigotes from the same tissue, is shown from a single experiment that is representative of 3 independent experiments. **B**) Expression of NOS2 mRNA in spleens of uninfected (Un) and 6-week *L. donovani* infected (Inf) mice and hamsters (n = 6 per group). The mean and standard error of the mean (SEM) of the fold-increase of NOS2 mRNA relative to BHK cells, determined by real time RT-PCR in 2 experiments is shown. **C**) Arginase activity in spleens of uninfected (Un) and 6-week *L. donovani* infected (Inf) mice and hamsters (n = 6 per group). The mean and standard deviation (error bars) of the tissue arginase activity, determined by assay of urea production, is shown from a single experiment that is representative of 3 independent experiments. **D**) Expression of arg1 mRNA in spleens of uninfected (Un) and 4-week *L. donovani* infected (Inf) mice and hamsters. The mean and standard deviation (error bars) of the fold-increase of arg1 mRNA relative to BHK cells, determined by real time RT-PCR in groups of 7 animals, is shown from a single experiment that is representative of 2 independent experiments. **E**) Expression of *L. donovani* arginase mRNA in spleens of 4-week *L. donovani* infected mice and hamsters. The mean and standard deviation (error bars) of the fold-increase of *L. donovani* arg1 mRNA relative to BHK cells, determined by real time RT-PCR in groups of 7 animals, is shown from a single experiment that is representative of 2 independent experiments. **F**) Log_10_ ratio of hamster arg1 mRNA to *L. donovani* arginase mRNA (open bars) and log_10_ ratio of hamster arg1 mRNA to hamster NOS2 mRNA (filled bars) in the spleens of 4-week *L. donovani* infected mice and hamsters. The mRNA expression was determined by real time RT-PCR in groups of 7 animals and used to calculate the mean and SD of the log ratios. The ratios were calculated from the raw data shown in Figs. 1B, 1D, and 1E. The statistical significance of differences in each of the panels is identified by asterisks (** p<0.01; ***, p<0.001).

Systemic infection of hamsters with *L. donovani* resulted in progressive, lethal VL [47]–[49]. This contrasted sharply with *L. donovani* infection in the mouse, which did not cause lethal disease and had a significantly lower parasite burden compared to hamsters infected with the same number of parasites (Fig. 1A). We demonstrated previously that in the hamster model of VL, there was impaired macrophage activation and parasite killing [49]. This was accompanied by transcriptionally-mediated low NOS2 expression and NO production in the infected hamster compared to the infected mouse ([48], [49] and Fig. 1B). We hypothesized that the low NO production would favor default toward an arginase-dominated metabolism of arginine at the site of infection. Indeed, the low expression of NOS2 in the hamster spleen was accompanied by high splenic arginase activity, measured by enzymatic conversion of arginine to urea (Fig. 1C), and this pattern of high arginase activity/NOS2 expression was reversed in the infected mouse spleen (Figs. 1B and 1C).

**Figure 2:**
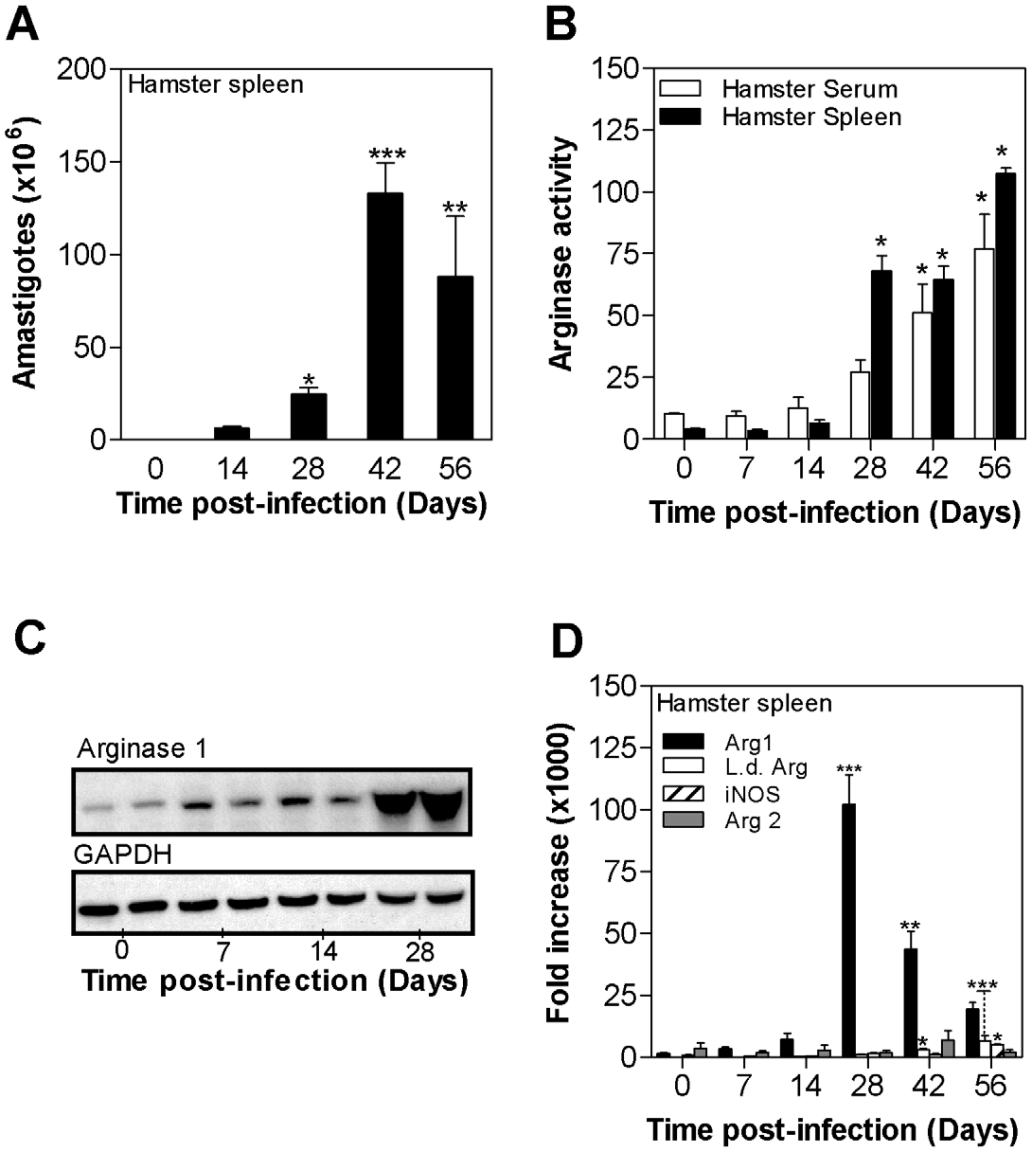
Kinetics of arginase expression in spleen tissue during progressive visceral leishmaniasis. A ) Parasite burden in the spleens of hamsters (n = 6 per group) infected with *L. donovani* for 14, 28, 42, and 56 days. The mean and SEM (error bars) of the parasite burden, determined by luminometry and interpolation from an amastigote standard curve, is shown from a single experiment that is representative of 2 independent experiments. **B**) Arginase activity in the serum (open bars) and spleen tissue (filled bars) of uninfected hamsters (Day 0) and hamsters infected with *L. donovani* (n = 6 per group) for 7, 14, 28, 42, and 56 days. The mean and SD of the tissue arginase activity, determined by assay of urea production, is shown. **C**) Hamster arg1 protein expression determined by western blot in spleens of control hamsters (0 days post-infection) and hamsters infected with *L. donovani* for 7, 14, and 28 days. The expression of GAPDH is shown as a control for protein loading. Each lane contains splenic lysate from a single hamster, with two lanes per time point. The anti-arginase antibody did not react with parasite arginase by immunoblot. **D**) Time course of expression of hamster arg1 mRNA (filled bars), *L. donovani* arginase mRNA (empty bars), hamster NOS2 (iNOS) mRNA (hatched bars), and hamster arg2 mRNA (hatched bars) in spleens of control hamsters (0 days post-infection) and hamsters infected with *L. donovani* for 7, 14, 28, 42, and 56 days. The mean and standard deviation (error bars) of the fold-increase of arginase mRNA relative to BHK cells, determined by real time RT-PCR in groups of 6 animals, is shown from a single experiment that is representative of 3 independent experiments. The statistical significance of differences in each of the panels is identified by asterisks (*, p<0.05; **, p<0.01; ***, p<0.001).

To determine the source of the increased arginase activity we first analyzed the expression of parasite and host arginase mRNAs. We cloned the hamster arg1 and arg2 cDNAs and the *L. donovani* arginase cDNA and measured expression of these cDNAs by real time RT-PCR because the biochemical measurement of arginase did not discriminate between the mammalian isoforms and parasite arginase. Hamster arg2 expression was not increased in infected compared to normal tissue (see Fig. 2D), but there was a striking increase in hamster arg1 mRNA in the spleens of hamsters with progressive VL compared to uninfected hamsters and uninfected or infected mice (Fig. 1D). Additionally, infection did not upregulate splenic arg1 mRNA expression in mice (Fig. 1D). The expression of *L. donovani* arginase transcripts paralleled the splenic parasite burden in infected mice and hamsters (Fig. 1E), but was a relatively minor contributor to the overall expression of arginase in hamsters (Fig. 1F; Fig. 2B). When the expression of parasite arginase and host NOS2 was compared to host arg1 a striking difference between mice and hamsters was evident. The level of hamster arg1 was more than 1000-fold greater than the level of parasite arginase in infected spleen tissue, whereas the ratio was inverted in the infected mouse spleen with parasite arginase being greater than host arg1 expression (Fig. 1F, open bars). Similarly, the host arg1 to NOS2 ratio was high in the infected hamster spleen, but the ratio was reversed in the infected mouse spleen (Fig. 1F, filled bars).

Kinetics studies revealed a sharp increase in the visceral parasite burden of L. donovani infected hamsters relatively late in the course of infection (Fig. 2A) that was accompanied by increased arginase activity in the serum and spleen (Fig. 2B). Dramatically increased expression of arg1 protein (Fig. 2C) and mRNA (Fig. 2D) was also evident in the spleen of infected hamsters. Unexpectedly, we found discordance between the level of hamster arg1 mRNA expression and arg1 protein expression and arginase enzyme activity late in the course of infection; after the peak at 28 days post-infection, the mRNA expression decreased but the level of protein and enzyme activity remained high. Hamster arg2 mRNA was not expressed in the spleen throughout the course of infection, and hamster NOS2 mRNA was increased only slightly at day-56 of infection (Fig. 2D).

**Figure 3:**
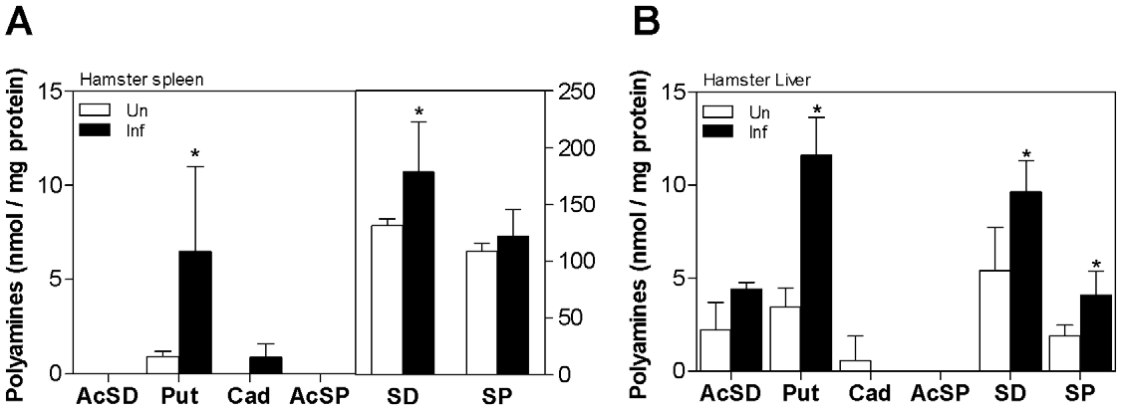
Polyamine content in spleen and liver tissue in *L. donovani* infected hamsters. The concentration of polyamines in spleen (**A**) and liver (**B**) tissue samples from groups of 6 uninfected hamsters (open bars) and 56 day infected hamsters (filled bars) is expressed as the mean and SD (error bars) of nmol polyamine per mg protein. The data shown are from a single experiment that is representative of 2 independent experiments. The statistical significance of differences in each of the panels is identified by an asterisk (*, p<0.05).

Several polyamines are end-products of arginine metabolism through the action of arginase and ornithine decarboxylase [56]–[58]. We confirmed the downstream effect of increased arg1 expression in hamsters with VL by demonstrating increased putrescine and spermidine in infected compared to uninfected spleen tissue (Fig. 3A), and increased putrescine, spermine, and spermidine in the infected liver (Fig. 3B). In infected hamsters there was no increase in the tissue content of acetylspermine and acetylspermidine, or cadaverine, which is a product of lysine metabolism. The tissue polyamine content in the spleen and liver of mice was relatively low compared to the hamster tissue, and it was not increased in infected compared to uninfected mice (Fig. S1).

### Cytokines that promote alternative macrophage activation are upregulated late in the course of VL

**Figure 4:**
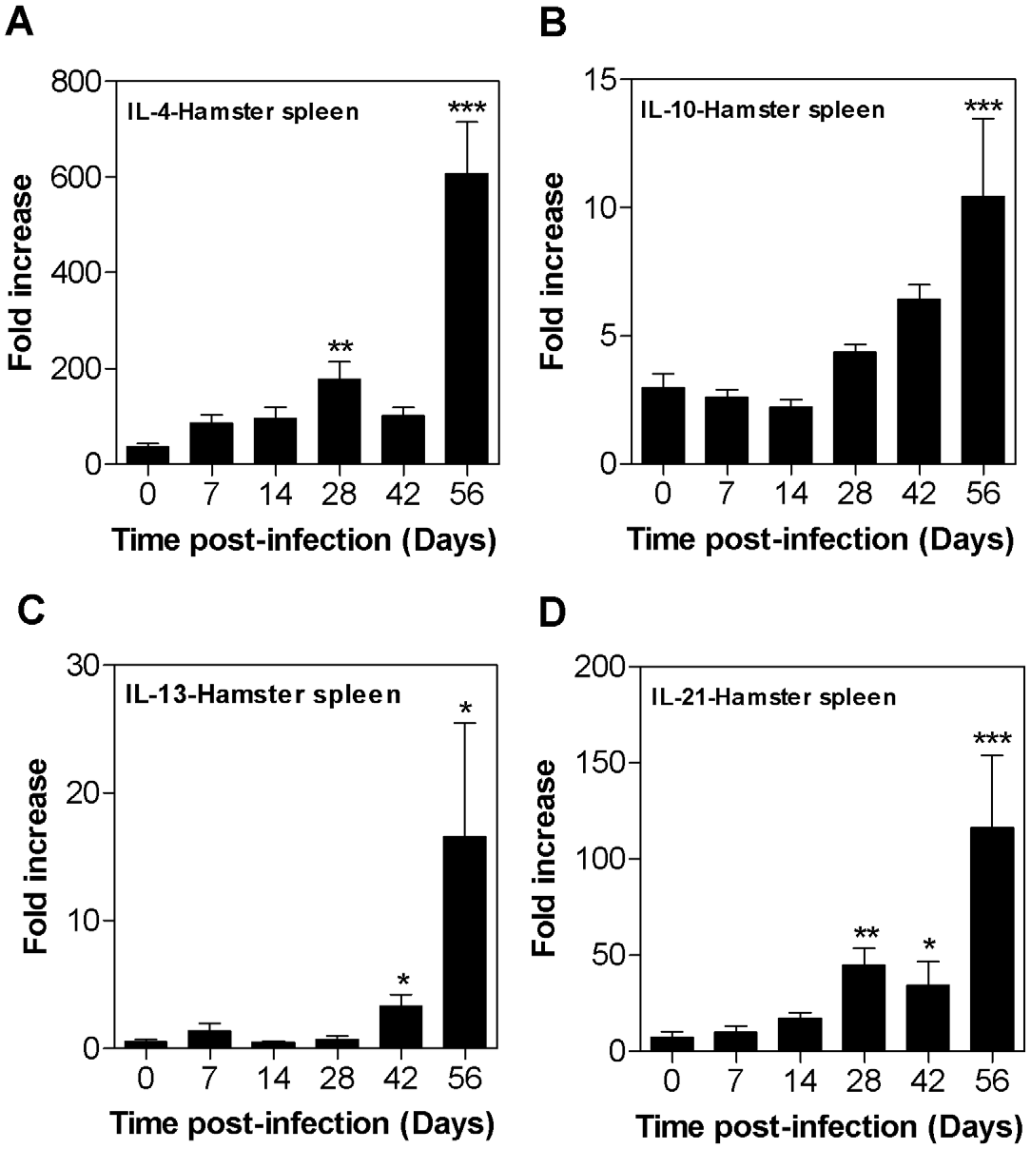
Kinetics of cytokine expression in spleen tissue during progressive visceral leishmaniasis. Expression of hamster IL-4 (**A**), IL-10 (**B**), IL-13 (**C**), and IL-21 (**D**) mRNAs in the spleens of control hamsters (0 days post-infection) and hamsters infected with *L. donovani* for 7, 14, 28, 42, and 56 days. The mean and standard deviation (error bars) of the fold-increase of cytokine mRNA relative to BHK cells, determined by real time RT-PCR in groups of 6 animals, is shown from a single experiment that is representative of 2 independent experiments. The statistical significance of differences in each of the panels is identified by asterisks (*, p<0.05; **, p<0.01; ***, p<0.001).

**Figure 5:**
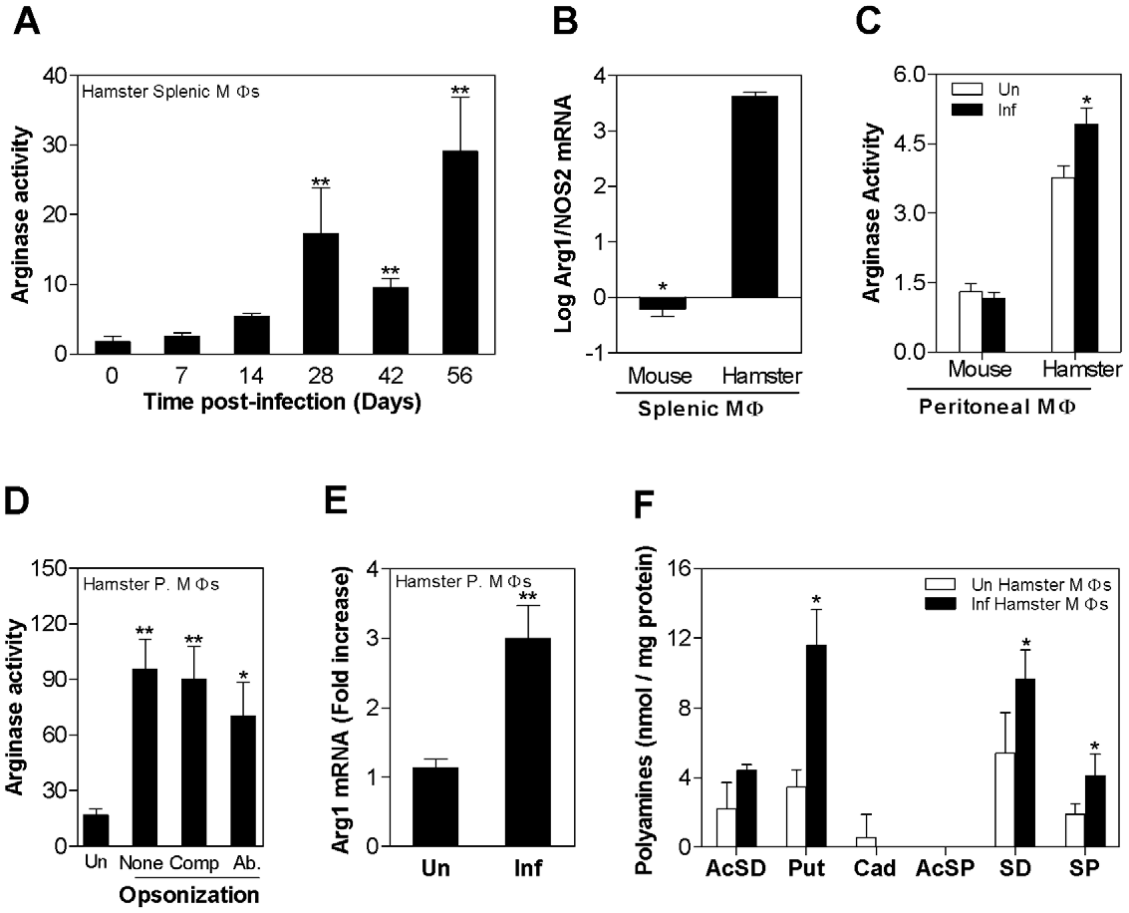
Arg1 expression and polyamine content in *L. donovani* infected macrophages. **A**) Arginase activity in macrophages isolated by adherence from single-cell suspensions from the spleens of control hamsters (0 days post-infection) and hamsters infected with *L. donovani* for 7, 14, 21, 42, and 56 days. The mean and SD (error bars) of the arginase activity in 100,000 cells, determined by assay of urea production, is shown from a single experiment that is representative of 2 independent experiments. **B**) Log_10_ ratio of host arg1 to NOS2 mRNA in splenic macrophages of 4-week *L. donovani* infected mice and hamsters. The mRNA expression was determined by real time RT-PCR in groups of 5 animals and used to calculate the log ratios, shown as the mean and SD (error bars) from a single experiment that is representative of 2 independent experiments. The ratios were determined from the following raw data: Arg1 mRNA fold-increase with reference to the BHK cell calibrator (mean ± SD): uninfected mice, 5.7±5.9; uninfected hamster, 3,367±2,858, infected mice, 8.5±10; infected hamster, 131,984±56,407; iNOS mRNA fold-increase with reference to BHK cell calibrator (mean ± SD): uninfected mice, 133.34±182.4; uninfected hamster, 1.03±0.3, infected mice, 2,984±4,535; infected hamster, 19.56±10.87). **C**) Arginase activity in peritoneal macrophages isolated from mice and hamsters that were uninfected (open bars) or infected in vitro with *L. donovani* for 48 hrs (filled bars). The mean and SD (error bars) of the arginase activity, determined by assay of urea production, is shown from a single experiment that is representative of 2 independent experiments. **D**) Arginase activity in hamster peritoneal macrophages that were uninfected (Un) or infected in vitro with *L. donovani* (1∶5 macrophage:parasite ratio). Parasites were either unopsonized (None), opsonized with normal fresh complement-containing hamster serum (Comp), or opsonized with freeze-thawed hamster serum containing anti-Leishmania antibody (Ab). The mean and standard deviation (error bars) of the arginase activity in 200,000 cells of 6 different samples determined by assay of urea production, is shown from a single experiment that is representative of 2 experiments. Statistical comparisons are made to the control group. **E**) Expression of hamster arginase mRNA in hamster peritoneal macrophages that were uninfected (Un) or infected in vitro with *L. donovani* stationary-phase promastigotes for 24 hrs (Inf). The mean and SEM (error bars) of the fold-increase of arg1 mRNA relative to BHK cells, determined by real time RT-PCR in groups of 6 samples per experiment, is shown as data pooled from 4 independent experiments. **F**) The concentration of polyamines in uninfected (open bars) and 48-hour in vitro infected hamster peritoneal macrophages (filled bars) (n = 6 per group) is expressed as the mean and SD (error bars) of nmol polyamine per mg protein. The data shown are from a single experiment that is representative of 2 independent experiments. The statistical significance of differences in each of the panels is identified by asterisks (*, p<0.05; **, p<0.01; ***, p<0.001).

The dominant expression of hamster arg1 in progressive VL suggested that IL-4, IL-10, IL-13, or IL-21, or a combination of these cytokines, which are known to promote alternative macrophage activation (reviewed in [36]), might be driving the expression of arg1. Since the type 2 cytokines induce arginase expression through a STAT6-dependent pathway, we first investigated whether there was evidence of STAT6-inducing activity in the serum and spleens of hamsters with VL. Using a STAT6 reporter assay, we found that there was a significant increase in STAT6-inducing activity in the serum (56% increase; *p*<0.05) obtained from hamsters with active VL (56 days post-infection) compared to uninfected controls. These findings led us to consider that the type 2 cytokines might contribute to the increased expression of arg1 in VL. After systemic infection with *L. donovani*, a relatively silent phase during the first ∼3 weeks of infection was followed by increased expression of IL-4, IL-10, IL-13, and IL-21 (Fig. 4). The increase in these cytokines was coincident with, but did not appear to precede, the dramatic increase in parasite burden and arginase activity (see Figs. 2 and 5A).

### *L. donovani* induces arg1 expression in infected macrophages

The increase in splenic arginase activity over the course of infection was paralleled by a similar increase in arginase activity in macrophages isolated from the spleens of hamsters infected with *L. donovani* (Fig. 5A). The reciprocal expression of arg1 and NOS2 mRNAs was also observed in splenic macrophages isolated from infected mice and hamsters. Splenic macrophages isolated from 4-week infected hamsters had a high arg1 to NOS2 ratio, but this ratio was reversed in splenic macrophages isolated from 4-week infected mice (Fig. 5B). Furthermore, resident peritoneal macrophages from hamsters with VL showed increased arginase activity compared to peritoneal macrophages from uninfected animals, but arginase activity in peritoneal macrophages from mice infected with the same number of parasites was not increased (Fig. 5C). An increase in *L. donovani*-induced arginase activity (Fig. 5D) and arg1 mRNA (Fig. 5E) was also evident in hamster peritoneal macrophages infected in vitro with *L. donovani* promastigotes. Opsonization of *L. donovani* promastigotes with either fresh hamster serum containing complement or hamster serum containing anti-*Leishmania* antibodies did not influence the parasite-mediated induction of arginase activity (Fig. 5D). Congruent with the findings of increase polyamine synthesis in infected hamster tissues (see Fig. 3), the downstream effect of the increased arginase activity in infected hamster macrophages was evident by increased polyamine (putrescine, spermidine, and spermine) synthesis compared to uninfected cells (Fig. 5F).

### Parasite-induced arg1 requires de novo protein synthesis but not parasite internalization

**Figure 6:**
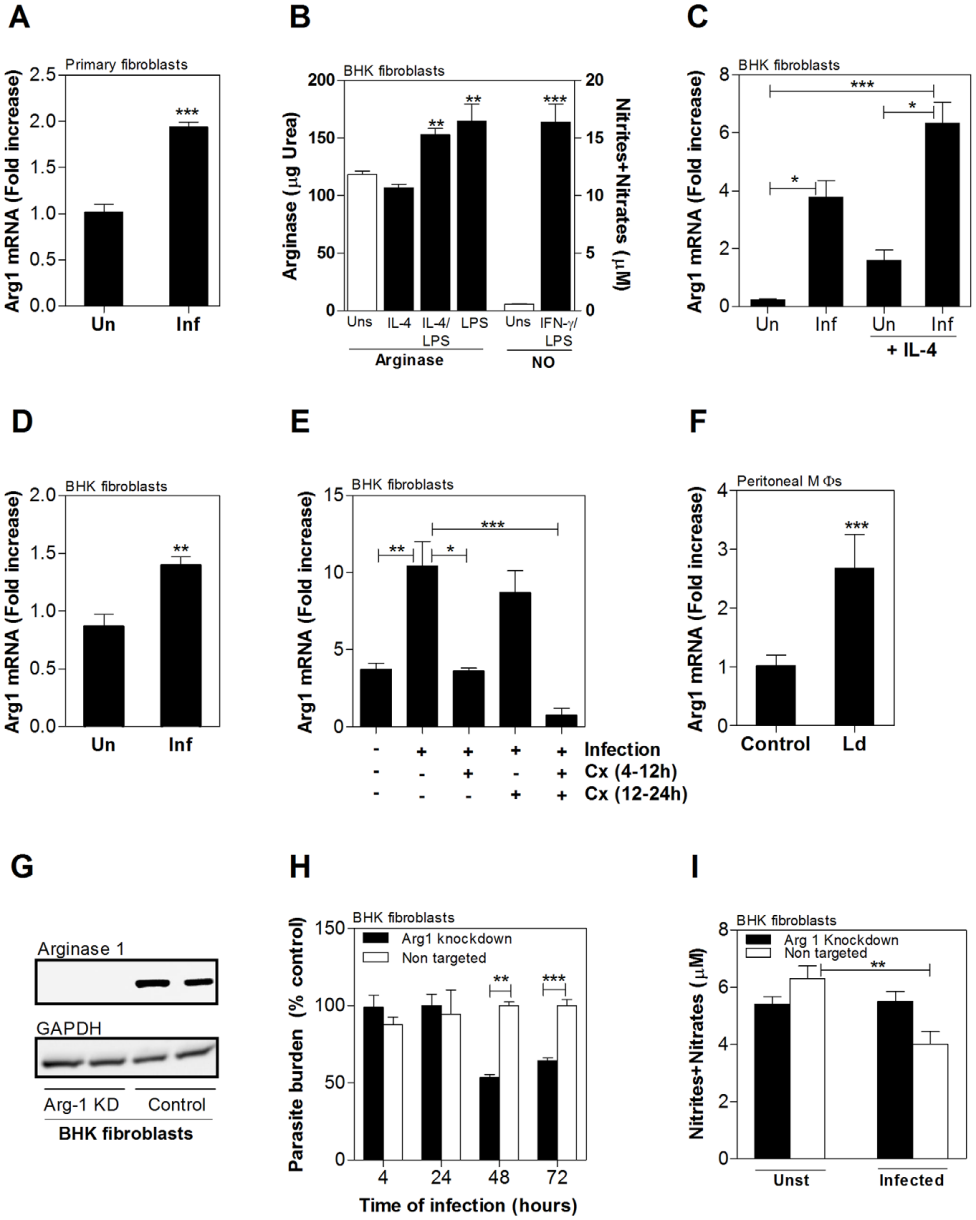
Parasite-induced host arg1 expression impairs macrophage anti-leishmanial activity. **A**) Expression of hamster arginase mRNA in primary hamster splenic fibroblasts that were uninfected (Un) or infected in vitro with *L. donovani* stationary-phase promastigotes for 24 hrs (Inf). The mean and standard deviation (error bars) of the fold-increase of arg1 mRNA relative to normal BHK cells, determined by real time RT-PCR in groups of 6 animals, is shown from data pooled from 3 independent experiments. **B**) Arginase activity and NO production in BHK fibroblasts. Arginase activity was determined stimulated with IL-4 or IFN- γ respectively. Arginase activity was determined in BHK cells that were unstimulated (Uns) or stimulated for 48h with IL-4 (10% v/v), LPS (1 µg/mL), or both. The mean and standard deviation (error bars) of the arginase activity of 6 different stimulated samples compared to that of non stimulated cells determined by assay of urea production, is shown from a single experiment that is representative of 2 experiments. NO production was estimated by the measurement of nitrites + nitrates in supernatants of unstimulated cells (Uns) and cells stimulated with IFN-γ (10% v/v of hamster recombinant IFN-γ supernatants) plus 1 µg/mL LPS for 48h. The mean and standard deviation (error bars) of the nitrites/nitrates of 6 different samples determined by Griess assay is shown from a single experiment that is representative of 2 experiments. **C**) Expression of hamster arginase mRNA in hamster BHK cells that were uninfected (Un) or infected in vitro with *L. donovani* stationary-phase promastigotes for 24 hrs (Inf) and cultured with or without recombinant hamster IL-4 (10% v/v supernatant) or sham supernatant. The mean and standard deviation (error bars) of the fold-increase of arg1 mRNA relative to BHK cells, determined by real time RT-PCR in groups of 6 animals, is shown from data pooled from 3 independent experiments. **D**) Expression of hamster arginase mRNA in hamster BHK fibroblasts that were uninfected (Un) or infected in vitro with *L. donovani* tissue-derived amastigotes for 24 hrs (Inf). The mean and standard deviation (error bars) of the fold-increase of arg1 mRNA relative to BHK cells, determined by real time RT-PCR in groups of 6 animals, is shown from data pooled from 2 independent experiments. **E**) Effect of cycloheximide (CHX) on parasite-induced hamster arg1 mRNA expression in BHK cells was determined by exposing cells to CHX (20 µg/mL) early (4–12 hrs) and/or late (12–24 hrs) during a 24-hr *L. donovani* infection. The mean and SD (error bars) of the fold-increase of arg1 mRNA relative to BHK cells, determined by real time RT-PCR in groups of 6 samples, is shown from data from a single experiment that is representative of 2 independent experiments. **F**) Induction of expression of hamster arg1 in peritoneal macrophages by soluble parasite factors was determined by culturing hamster peritoneal macrophages with stationary phase promastigotes (1∶10 ratio) separated by a 0.4 µ pore size membrane (Falcon). The mean and SEM (error bars) of the fold-increase of arg1 mRNA relative to BHK cells, determined by real time RT-PCR in groups of 6 animals, is shown from data pooled from 3 independent experiments. **G**) miRNAi-mediated arg1 knockdown in BHK cells. Arg1 protein in BHK cells stably transfected with a miRNAi vector targeting arg1 (Arg-1 KD) or stably transfected with a miRNAi vector coding a control sequence (Control) determined by Western blot using a specific polyclonal antibody raised against hamster arg1. Representative of 2 different blots. **H**) Effect of miRNAi-mediated knockdown on parasite burden was determined in BHK cells that were transfected with a non-targeting miRNAi vector (control) or a vector specific to hamster arg1. The transfected cells were infected with *L. donovani* metacyclic promastigotes and the mean and standard deviation (error bars) of the parasite burden at 4, 24, 48 and 72 hrs post-infection, determined by luminometry, is shown from a single experiment that is representative of 2 independent experiments. There was no difference in parasite burden between the two groups at 4 and 24 hrs post-infection. **I**) Arg1 knockdown did not induce NO production in BHK cells infected with *L. donovani*. NO production in BHK cells stably transfected with a miRNAi vector targeting Arg1 (Arg1 knockdown) or transfected with an irrelevant miRNAi vector (Non targeted). The mean and standard deviation (error bars) of the nitrites/nitrates released in the supernatant of cells after 48h infection with *L. donovani* was determined by Griess assay. Data are from a single experiment that is representative of 3 independent experiments. (**p<0.001). The statistical significance of differences in each of the panels is identified by asterisks (*, p<0.05; **, p<0.01; ***, p<0.001).

We next determined if *de novo* protein synthesis was required for the parasite-induced increase in arg1 mRNA. To do this we established an in vitro infection model in the BHK hamster fibroblast cell line, since primary macrophages were killed by the protein synthesis inhibitor cycloheximide, even at very low doses, and no hamster macrophage cell lines were available. We first demonstrated that, like hamster macrophages (Fig. 5D), hamster primary splenic fibroblasts expressed arg1 in response to in vitro infection with *L. donovani* (Fig. 6A). Furthermore, we showed that BHK fibroblasts could be infected with *L. donovani* (confirmed by microscopical examination and co-localization with phagolysosomal staining; see Fig. S2), and were capable of metabolizing arginine through either the NOS2 or arginase pathways. BHK fibroblasts generated NO in response to classical activation stimuli (IFN-γ/LPS) and expressed arginase activity in response to LPS as was described for macrophages from murine rodents [62] (Fig. 6B). Surprisingly, IL-4 alone did not induce arginase activity in BHK fibroblasts. We next showed that like hamster macrophages, in vitro infection of BHK fibroblasts resulted in parasite-induced arg1 mRNA expression (Fig. 6C). The parasite-induced arg1 expression was amplified by concomitant exposure to exogenous recombinant hamster IL-4 (Fig. 6C). The induction of arg1 expression was also evident in BHK cells infected with spleen-derived purified amastigotes (Fig. 6D). Dose titration studies identified cycloheximide concentrations that effectively blocked protein synthesis but did not significantly reduce cell viability in BHK cells. Parasite-induced arg1 transcription was blocked by non-toxic concentrations of cycloheximide (CHX) when the CHX was present during the first 12 hrs of exposure to the *Leishmania* (Fig. 6E), indicating that early *de novo* synthesis of either an autocrine or paracrine protein, was required for arg1 mRNA expression. However, infected BHK cells did not express IL-4 mRNA (by real-time RT-PCR the C_T_ value for GAPDH was 22.9±0.09 and the C_T_ value for IL-4 was below the threshold limit of detection), and expressed very low basal levels of IL-13 that did not increase with infection (after correction for GAPDH expression the fold-increase relative to the BHK calibrator was 1.14 ± 0.16 for uninfected cells and 0.98±0.2 for infected cells; *p* = 0.16), suggesting that the parasite-induced arg1 expression was independent of type 2 cytokine synthesis. The parasite-induced transcription of arg1 did not require the internalization of parasites, since separation of promastigotes from hamster cells with a 0.4 µ membrane did not abrogate the parasite-induced increase in arg1 mRNA expression (Fig. 6F).

### Knockdown of host arg1 promotes parasite killing without enhanced NO production

Since arginase and NOS2 compete for the same substrate, arginine, we reasoned that inhibition of arginase might reverse the low NO production observed in activated hamster macrophages and lead to enhanced parasite killing. Treatment with the arginase inhibitor norNOHA resulted in a significant dose-dependent reduction in arginase activity (Fig. S3, panel A) and parasite burden (Fig. S3, panel B) in peritoneal macrophages infected in vitro, and a reduction in parasite burden in ex vivo cultured spleen cells isolated from infected hamsters (Fig. S3, panel C). However, we found that norNOHA demonstrated a dose-dependent killing of amastigotes purified from infected hamster spleens (Fig. S3, panel D) and axenically cultured *L. donovani* promastigotes (Fig. S3, panel E), suggesting that it was inhibiting parasite arginase. Therefore, to investigate the role of arginase in parasite replication without the potential confounding influence of parasite arginase activity, we used the BHK infection model to knockdown host arg1. Transfection of BHK cells with an arg1-specific miRNAi vector resulted in >90% reduction in arg1 mRNA expression (*p* = 0.002) and reduction of arg1 protein to a level undetectable by western blot (Fig. 6G). *L. donovani* infected BHK cells that expressed the arg1-specific miRNAi vector were found to have an equivalent parasite burden at 4 and 24 hrs post-infection but significantly reduced parasite burden at 48 hrs (*p*<0.01) and 72 hrs (*p*<0.001) post-infection compared to non-transfected cells or cells transfected with a non-targeting miRNAi construct (Fig. 6H). The approximately 50% decrease in parasite burden following arg1 knockdown suggests that either there is residual arginase expression (undetectable by western blot) that is sufficient to promote some parasite survival, or more likely that arginase is not the only determinant of parasite survival and replication in this model. This enhancement of parasite killing by knockdown of arg1 in BHK cells was not accompanied by greater parasite-induced NO production (Fig. 6I), even though BHK cells were fully capable of generating NO (see Fig. 5B). Thus it would appear that parasite-induced macrophage arg1 contributes to *L. donovani* replication through mechanisms other than reduction of NO production.

### STAT6 activation drives parasite-induced arg1 expression in VL

**Figure 7:**
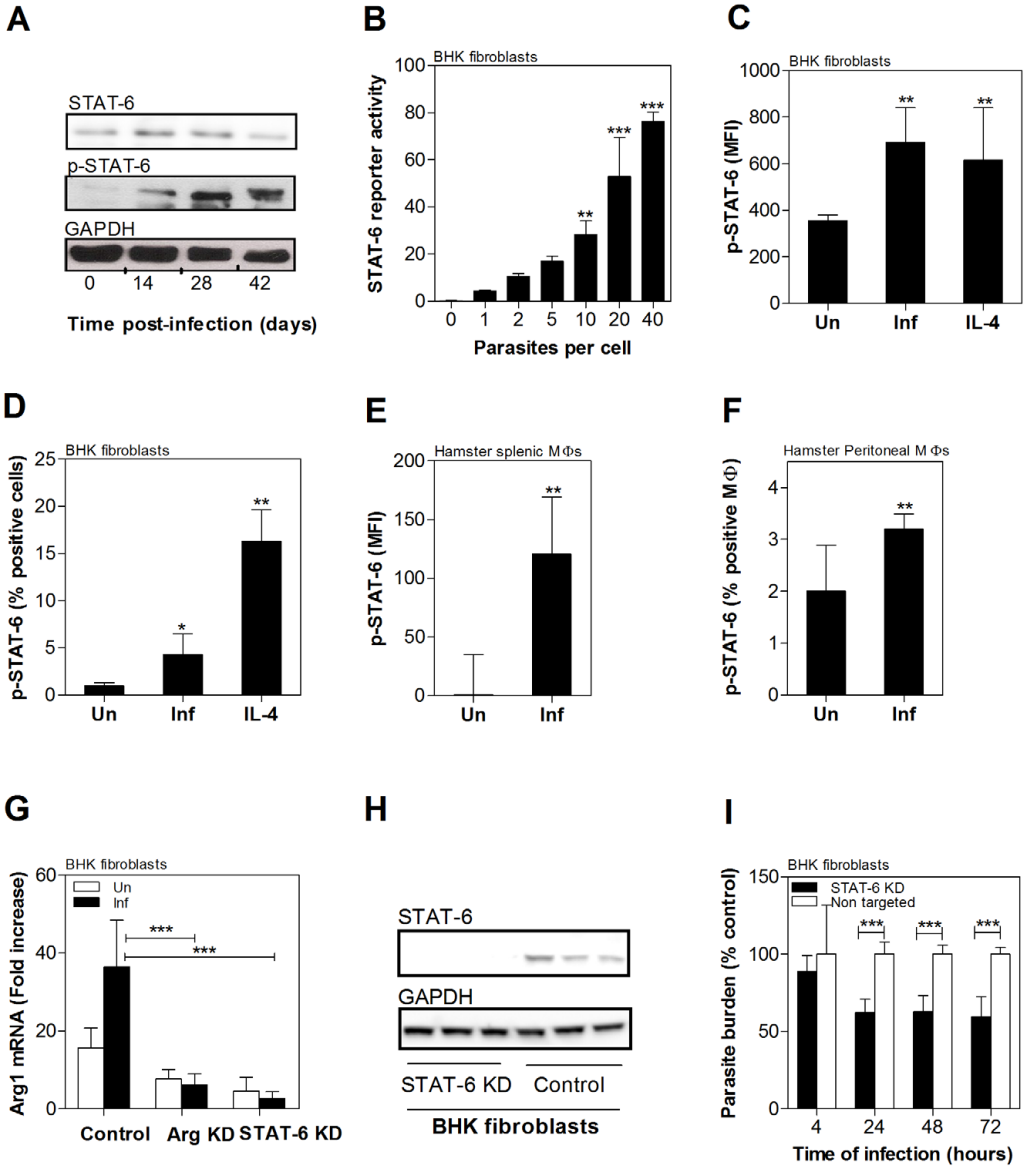
Role of parasite-induced STAT6 activation in host arg1 expression and *L. donovani* infection. **A**) Phosphorylated STAT6 in spleens of hamsters infected with *L. donovani* determined by immunoprecipitation followed by western blot of whole splenic lysates. The samples (before immunoprecipation) were probed with anti-STAT6 anti-GAPDH antibodies to confirm equivalent protein loading. The blot shown is from tissue from a single animal, representative of 4 independent experiments. **B**) Dose-dependent induction of STAT6 activation by *L. donovani* in BHK cells transfected with a STAT6-luciferase reported vector. Data are presented as the mean and standard deviation (error bars) of the relative light units in uninfected (0 parasites) cells and cells exposed to 1–40 parasites per cell over 48 hrs of culture. Shown is data from a single experiment that is representative of 2 independent experiments. **C-D**) Phosphorylation of STAT6 in hamster peritoneal macrophages exposed to *L. donovani* (10 parasites per cell) or IL-4 (10% v/v supernatants) measured as fold-increase of mean fluorescence intensity (MFI) (**C**) or percent positive cells (**D**) by flow cytometry. Data are presented as the mean and standard deviation (error bars) and are from a single experiment that is representative of 2 independent experiments. **E–F**) Phosphorylation of STAT6 in hamster BHK cells exposed to *L. donovani* (10 parasites per cell) measured as fold-increase of mean fluorescence intensity (MFI) (**E**) or percent positive cells (**F**) by flow cytometry. Data are presented as the mean and standard deviation (error bars) and are from a single experiment that is representative of 2 independent experiments. **G**) Effect of miRNAi-mediated STAT6 knockdown on arg1 and STAT6 mRNA expression. BHK cells were transfected with a non-targeting miRNAi vector (control) or a vector specific to hamster STAT6 and the expression of arg1 and STAT6 mRNA determined by real time RT-PCR in uninfected (open bars) and 24-hr infected cells (filled bars). The data are presented as the mean and standard deviation (error bars) of the fold-increase of arg1 mRNA relative to BHK cells, determined by real time RT-PCR from a single experiment, representative of 2 independent experiments. **H**) miRNAi-mediated STAT-6 knockdown in BHK cells. The expression of STAT-6 protein in BHK cells stably transfected with a miRNAi vector targeting STAT-6 (STAT-6 KD) or transfected with a miRNAi vector coding a control sequence (Control) was determined by western blot using an anti-STAT6 antibody. An anti-GAPDH antibody was used to confirm equivalent protein loading. Data are from a single blot representative of 2 independent experiments. **I**) Effect of miRNAi-mediated STAT6 knockdown on parasite burden was determined in BHK cells that were transfected with a non-targeting miRNAi vector (control) or a vector specific to hamster STAT6. The transfected cells were infected with *L. donovani* metacyclic promastigotes and the mean and standard deviation (error bars) of the parasite burden at 4, 24, 48, and 72 hrs post-infection, determined by luminometry, is shown as data pooled from 2 independent experiments. There was no difference in parasite burden between the two groups at 4 hrs post-infection. The statistical significance of differences between groups in each of the panels is identified by asterisks (*, p<0.05; **, p<0.01; ***, p<0.001).

The expression of phosphorylated STAT6 was increased in the spleen tissue of hamsters over the course of in vivo infection with *L. donovani* (Fig. 7A). To dissect the role of STAT6 in *L. donovani*-induced arg1 expression we used the in vitro infection model of the BHK hamster fibroblast cell line as described above. Using an in vitro reporter assay we found that exposure of hamster fibroblasts to metacyclic *L. donovani* promastigotes activated STAT6 in a dose-dependent manner (Fig. 7B). The STAT6 activation was not influenced by parasite opsonization with complement-containing serum (Fig. S4). Parasite-induced STAT6 phosphorylation was further confirmed by flow cytometry in infected BHK cells (Figs. 7C and 7D), in splenic macrophages isolated from hamsters with VL (Fig. 7E), and in hamster peritoneal macrophages infected in vitro with *L. donovani* promastigotes (Fig. 7F). The percent of macrophages that showed parasite-induced STAT-6 phosphorylation was relatively lower than the percent positive activated by IL-4 (Fig. 7D). Knockdown of hamster STAT6 in BHK cells using miRNAi resulted >90% decrease in mRNA expression (Fig. 7G) and reduction of STAT6 protein to a level undetectable by western blot (Fig. 7H). The significance of parasite-induced STAT6 activation was confirmed by showing a 92% reduction in arg1 mRNA expression when STAT6 was knocked-down (similar to the reduction obtained with arg1-specific knockdown) compared to a control non-targeting miRNAi (*p*<0.001; Fig. 7G) and enhanced control of intracellular parasite replication in BHK cells (equivalent infection after 4 hrs but significantly reduced parasite burden at 24, 48, and 72 hrs post-infection compared to non-transfected cells or cells transfected with a non-targeting miRNAi construct (*p* = 0.001; Fig. 7H). Collectively, these data indicate that parasite-induced STAT6 activation drives expression of host arg1, which in turn contributes to intracellular parasite replication and/or survival.

## Discussion

We initiated these studies because there is a deficit in our understanding of the molecular and cellular determinants underlying the pathogenesis of VL. We used a hamster model of VL because the clinicopathological features of this model closely mimic active human VL, and insights gained from this model may enhance our understanding of the immunopathogenesis of human disease. In the studies we present here we identified a program of alternative macrophage activation that is evident in the spleens of hamsters with VL, and in *L. donovani* infected macrophages. Characteristic of this phenotype is the dominant expression of host arg1 over NOS2 in infected hamster spleen tissue and macrophages. Here we show that *L. donovani* induces host arg1 expression through a mechanism that involves parasite-induced STAT6 activation, but different from the prevailing paradigm of alternative activation in cutaneous leishmaniasis [42]–[44], occurs even in the absence of a polarized type 2 cytokine response.

The tissue-level dominance of arginase over NOS2-mediated arginine metabolism is driven by increased host arg1 but not arg2 transcription. Furthermore, hamster arg1, which is expressed by macrophages in the infected spleen, is dominant over the level of parasite arginase even though parasite arginase expression increases throughout the course of infection. This contrasts sharply with what we found in the murine model of non-progressive *L. donovani* infection where host arg1 expression is not dominant, and parasite arginase appears to have a relatively greater contribution to the total arginase activity. Similarly, in the murine model of *L. major* infection, parasite arginase contributes significantly to the overall cellular arginase activity [63]. While it is logical that *L. donovani* arginase contributes to the pathogenesis of VL, our data suggest that it has a significantly lesser role than host arg1. Quantification of its contribution to the pathogenesis of VL in this model will require studies using an enzyme inhibitor selective for parasite arginase or arginase-deficient parasites.

The consequences and significance of the increased host arg1 expression in the progression of VL is underscored by several findings. First, during progressive infection there was an increase in tissue polyamines, which are end products of arginase- and ornithine decarboxylase-mediated metabolism of arginine. Polyamines, synthesized by the parasite or scavenged from host cells through uptake receptors [45], promote *Leishmania* growth [46]. The importance of polyamines is underscored by their critical role in the growth of a number of other protozoa, including *Trypanosoma brucei*, *T. cruzi*, *Toxoplasma* and *Plasmodium* [64]–[66]. Second, the increase in host arg1 transcription and enzyme activity paralleled the increase in the visceral parasite burden. This corroborates the findings in the murine model of *L. major* infection [42]–[44]. However, late in the course of hamster VL there is a relative decrease in the expression of arg1 mRNA but sustained arginase protein expression and enzyme activity, which is consistent with the previous notion that arg1 is also post-transcriptionally regulated [67]. Third, as noted above, we found that at the site of visceral infection (spleen) in hamsters there is dominant expression of arg1, such that the arg1 to NOS2 ratio in hamsters with progressive disease was thousands-fold greater than the ratio observed in mice, which are able to control the infection. The non-induced arg1 mRNA expression in the *L. donovani* infected mouse spleen in our study was different from the 4.8-fold increase reported in a recent study [68]. This difference may be due in part to infection with a different *L. donovani* strain (Indian vs. East African) and use of a much larger inoculum in the study by Biswas, et al. [68]. Nevertheless, the expression of arg1 in the *L. donovani* infected mouse is dramatically less than the increase observed in the hamster model of progressive VL. This dominant expression of arg1 in hamster VL lead to disease by production of parasite-promoting polyamines, or by driving arginine metabolism away from NOS2 and production of the anti-leishmanial effector molecule NO (which is already expressed at a low level). Fourth, and most significantly, targeted knockdown of host arg1 mRNA led to enhanced capacity to control intracellular parasite replication. Although other investigators demonstrated that chemical inhibition of arginase enhanced control of *Leishmania* infection [42], [44], we found that the arginase inhibitor nor-NOHA mediated a host-independent anti-parasitic effect, presumably by direct inhibition of parasite arginase (which was also recently demonstrated for *L. mexicana* arginase [69]), and therefore could not be used to distinguish an effect of host arginase independent of parasite arginase in our model. This increased control of infection mediated by targeted arg1 knockdown did not appear to be driven by enhanced production of NO by the isolated infected macrophage, suggesting that the parasite-induced arginase has a pathological effect through the increase in polyamines that promote parasite growth. This is consistent with the findings in *L. major* infection [44].

As stated previously, alternatively activated macrophages, as introduced by Gordon and colleagues, display a unique phenotype when activated in the presence of IL-4 or IL-13 [36], [37]. The gene expression profile of these macrophages includes reduced expression of NOS2, and increased expression of a number of unique genes, including arg1, which are transcriptionally activated by IL-4 through a STAT6-dependent mechanism [70], [71]. STAT6 is considered to be the central regulator of alternative macrophage activation [36]. While STAT6 may be activated by other stimuli such as IL-15, platelet-derived growth factor, kit ligand, and leptin, the canonical pathway for STAT6 activation is through IL-4 or IL-13 (reviewed in [72]). With this understanding, it is not surprising that the prevailing paradigm is that alternative activation of macrophages during murine *L. major* infection is a downstream effect of the dominant Th2 polarization and type 2 cytokine production seen in this model [42]–[44]. Indeed, the IL-4/IL-13/IL-4Rα/STAT6 signaling pathway has a well-established role in the pathogenesis of cutaneous *L. major* and *L. mexicana* infection in mice [73], [74]. However, these studies in the murine model focused on the effects of STAT6 deficiency in the T cell compartment without addressing the role of STAT6 in macrophages [74], [75]. We found that exposure of macrophages or fibroblasts to *L. donovani* led to activation of STAT6 (measured using an in vitro reporter assay, flow cytometry of infected macrophages and fibroblasts, and western blotting of phospho-STAT6) even in the absence of T cell signals or exogenous type 2 cytokines. The parasite-induced host arg1 expression was completely abolished by miRNAi-mediated knockdown of STAT6, and interruption of this pathway either by STAT6 or arg1 knockdown enhanced the control of intracellular parasite replication.

Although it has not been thoroughly studied, fibroblasts can be polarized by exposure to IL-4, resulting in STAT6 activation [76] and expression of arginase and other markers that are expressed by alternatively activated macrophages [77], [78]. *Leishmania* are known to infect fibroblasts [79], [80], and stromal cells are increasingly being recognized as modulators of host defense (reviewed in [81]). The finding of arg1 expression in splenic fibroblasts from hamsters with VL, and in fibroblasts infected in vitro with *L. donovani,* suggests that the “alternatively activated” phenotype extends to splenic stromal cells in VL, which are likely to contribute to the pathogenesis of the disease. The fact that IL-4 alone did not induce arginase activity in BHK fibroblasts supports the notion that the *Leishmania*-induced arg1 is driven through an IL-4-independent pathway in this cell.

Although early *de novo* synthesis of either an autocrine or paracrine protein was required for arg1 mRNA expression, this yet to be identified factor did not appear to be IL-4 or IL-13 since there was no increase in endogenous expression of these cytokines in the in vitro infected cell culture where parasite-induced STAT6 activation and arg1 expression were evident. This is not to say that signaling through IL-4/IL-13/IL-4Rα/STAT6 has no role in the pathogenesis of VL. The addition of exogenous IL-4 to the in vitro infection model clearly amplified the parasite-induced arg1 expression, and our in vivo data suggest that the prominent type 2 cytokine expression that is evident late in the course of VL serves to amplify arg1 expression and the alternative activation phenotype, and thus contributes to the relentlessly progressive infection. In human visceral and cutaneous leishmaniasis, there is increased expression of the type 2 cytokines at the site of chronic and severe infection [13], [14], [23], [82]–[84], but the role of these cytokines in the pathogenesis of human infection has not been fully defined. Other signaling molecules could also interface with this pathway to influence arg1 expression. A number of co-activator proteins, including, p100, CBP/p300, SRC-1, RNA pol II, PU.1, and C/EBP, are recruited with STAT6 to form a complex enhancer element in the promoter to initiate in arg1 transcription [85]–[87]. Of particular note, C/EBPβ may be activated by the IL-10/STAT3 pathway [88], [89]. The potential for synergistic interaction of the IL-4/IL-13/STAT6 and IL-10/STAT3 pathways was not fully appreciated until the recent work of Biswas, et al, who found that IL-10 expressed in the spleens of mice infected with *L. donovani* induced the upregulation of IL-4Rα, which was required for arg1 expression [68]. Identification of co-activators and additional pathways that contribute to STAT6-dependent *L. donovani*-induced arg1 expression in this model of progressive VL is currently under investigation.

A number of host factors other than type 2 cytokines can induce a macrophage activation profile that overlaps the classic IL-4- and IL-13-induced alternative macrophage activation [36], and could be contributing as an autocrine or paracrine factor in the *L. donovani*-induced arginase activity. These include the STAT3-activating cytokines IL-10, IL-6, and G-CSF [90], [91], TGF-β [46], cAMP [92], and PGE2 [93]. Of these, IL-10 is particularly noteworthy for several reasons: (1) IL-10 is increased in patients [13], [14], [19], [22]-[25] and hamsters with VL ([60] and current work), (2) in vitro neutralization of IL-10 in cultures of peripheral blood mononuclear cells from patients with VL led to recovery of suppressed Th1 responses [12], [26], (3) parasite replication in human macrophages was enhanced by exposure to recombinant IL-10 [94], (4) neutralization of IL-10 in serum from patients with VL cultured with *in vitro* infected macrophages, or in cultured splenic aspirates of patients with VL resulted in reduced parasite burden [19], [27], and (5) IL-10 deficient mice show increased resistance to experimental *L. donovani* infection [5], [6]. As noted above, the recently described synergy between IL-4/STAT6 and IL-10/STAT3 in the murine model of *L. donovani* infection [68] suggests that IL-10 could augment the STAT6-dependent arg1 expression in the hamster model.

The role of arginase in the pathogenesis of human VL is uncertain. Polarization of isolated human macrophages by exposure to IL-4 in vitro does not lead to upregulation of arginase activity or arg1 expression [95]. However, the presence of alternatively activated monocytes/macrophages and arginase expression has been found in some human disease states. Human filarial infection is associated with enhanced expression of a number of genes related to alternative activation in peripheral blood mononuclear cells, including arg1 [96]. Arginase activity and arg1 expression were also increased in peripheral blood mononuclear cells from patients following traumatic tissue injury [97]. The expression of arginase by myeloid cells in the human tumor microenvironment is well established [98], [99]. Studies of the role of AAMs and arginase in the pathogenesis of human VL are certainly warranted.

While alternatively activated macrophages have been described in a number of protozoan and metazoan infections (reviewed in [100], [101]), rarely has a parasite antigen or product been found to directly induce arginase in isolated macrophages. We found that STAT6 activation and arg1 transcription could be initiated through parasite-derived soluble factors and did not require parasite contact or internalization by the host cell. Stempin et al [39] found that the cruzipain antigen from *Trypanosoma cruzi* directly induced the expression of host arignase. Recently, a proteophosphoglycan produced by *L. mexicana* within the sand fly vector was shown to induce arginase activity in inflammatory macrophages and enhance intracellular parasite replication [102]. In addition, *Toxoplasma gondii* was found to activate STAT6 directly [103], and induce arginase through TLR-dependent, but STAT6-independent pathway [104]. Lastly, it was demonstrated that the Ym1, another marker of alternative activation, was induced in macrophages exposed to a helminth antigen [105]. Work is underway to identify the soluble *L. donovani* factor(s) that induce macrophage arg1.

Collectively, these data lead us to propose a new model in which ineffective classical macrophage activation in experimental VL, which is reminiscent of human VL, is associated with, and perhaps enables, the emergence of a dominant program of STAT6-dependent alternative macrophage (and fibroblast) activation with impaired control of parasite replication. The metabolism of arginine through the arginase-polyamine pathway not only redirects arginine away from the generation of NO by NOS2, but also favors the production of polyamines, which promote parasite replication. That the type 2 cytokines can drive alternative macrophage activation is without question [36], [106], however, the greatest significance of our findings is that *L. donovani* can activate macrophage and fibroblast STAT6 and induce arg1 expression without synthesis of endogenous IL-4 or IL-13 or stimulation by exogenous cytokines. We postulate that in progressive VL these cytokines serve as an amplification factor for macrophages that have already started down the alternative activation pathway through interaction with *L. donovani* or a soluble parasite factor or factors. This notion is supported by the dramatic increase in parasite burden that accompanies the type 2 cytokine response first evident several weeks into the course of infection. Further dissection of the pathway by which *L. donovani* drives host arg1 expression may identify unique pathogenic mechanisms and targets for therapeutic intervention.

## Supporting Information

Figure S1 **Polyamine content in spleen and liver tissue in** ***L. donovani*** **infected mice.** The concentration of polyamines in spleen and liver from groups of 5 uninfected mice (open bars) and 5 infected mice (filled bars) is expressed as the mean and standard deviation (error bars) of nmol polyamine per mg protein. The data shown are from a single experiment that is representative of 2 independent experiments. There were no statistically significant differences between the uninfected and infected tissue samples.
(TIF)

Figure S2 ***L. donovani*** **infection of BHK cells.** BHK cells were infected at 10∶1 ratio with *L. donovani promastigotes* for 4 hours and then the extracellular parasites were removed by washing 3 times with PBS and once with 0.1% trypsin/EDTA (Gibco) in PBS for 3 min at 37°C. After the last wash the BHK monolayer was detached with 0.25% trypsin and the cells collected by centrifugation at 400 x g for 5 min. The pelleted cells were adjusted to 200,000 cells/200 µL of culture medium, transferred to 4-well chamber slides, and incubated for 24 h at 37°C, 5% CO_2_. (A-B) Intracellular amastigotes were imaged at 40× magnification after nuclear labeling with 2 µg/mL Hoechst 33342 (Molecular Probes, Invitrogen) for 5 min. A BHK nucleus is shown by an arrowhead and amastigotes are identified by arrows. In some instances the amastigotes are oriented so that the kinetoplast DNA is clearly visible. (C) *L. donovani* amastigotes in BHK cells imaged at 100X magnification after staining with Hoechst 33342 and (D) 100 nM Lysotracker Red DND-99 (Molecular Probes, Invitrogen) to stain the phagolysosome. (E) Overlay of the Hoechst and Lysotracker Red stained images using the NIS-Elements Software (Nikon) to confirm the intraphagolysosmal location of the amastigotes.
(TIF)

Figure S3 **Anti-** ***Leishmania*** **activity of the inhibitor nor-NOHA (Nω-hydroxy-nor-Arginine).** (A) Arginase activity was measured in supernatants of *L. donovani*-infected hamster peritoneal macrophages after incubation with or without nor-NOHA for 48 h. The data is shown as the mean and standard deviation (error bars) of the arginase activity determined by assay of urea production in 100,000 cells. Statistical differences are shown between untreated and treated samples. (B) Number of amastigotes in *L. donovani*-infected hamster peritoneal macrophages after incubation with or without nor-NOHA for 48 h. The data is shown as the mean and standard deviation (error bars) of the number of parasites determined by luminometry. (C) Number of amastigotes in splenic macrophages isolated from hamsters at 15 days post-infection and untreated or treated *ex vivo* for 48 h with nor-NOHA. The data is shown as the mean and standard deviation (error bars) of the number of parasites determined by luminometry. (D) Number of amastigotes (purified from infected hamster spleen) after 24 h of *in vitro* culture with or without nor-NOHA (seeded at 100,000 amastigotes/100 µL). The data is shown as the mean and standard deviation (error bars) of the number of parasites determined by luminometry. (E) Number of promastigotes after 24h of *in vitro* culture with or without norNOHA (seeded at 100,000 amastigotes/100 µL). The data is shown as the mean and standard deviation (error bars) of the number of parasites determined by luminometry. The data shown for each of the panels is from a single experiment representative of at least 2 independent experiments. The statistical significance of differences between groups in each of the panels is identified by asterisks (*, p<0.05; **, p<0.01; ***, p<0.001).
(TIF)

Figure S4 **Effect of opsonization on** ***L. donovani*** **-induced STAT-6 activation.** BHK cells transfected with a STAT6-luciferase reported vector were exposed or not to unopsonized, or complement opsonized (fresh hamster serum), or heat killed L. donovani promastigotes**.** Data are presented as the mean and standard deviation (error bars) of the relative light units in uninfected (Un) cells and cells exposed to 10 parasites per cell over 48 hrs of culture. Shown is data from a single experiment that is representative of 2 independent experiments. The statistical significance of differences between uninfected and parasite-exposed groups is identified by asterisks (*, p<0.05).
(TIF)
