# Supplementary figures and images for: Progressive Visceral Leishmaniasis Is Driven by Dominant Parasite-induced STAT6 Activation and STAT6-dependent Host Arginase 1 Expression
Source: PLoS Pathog. 2012 Jan 19;8(1):e1002417. doi: 10.1371/journal.ppat.1002417 (PMC3261917; doi:10.1371/journal.ppat.1002417)

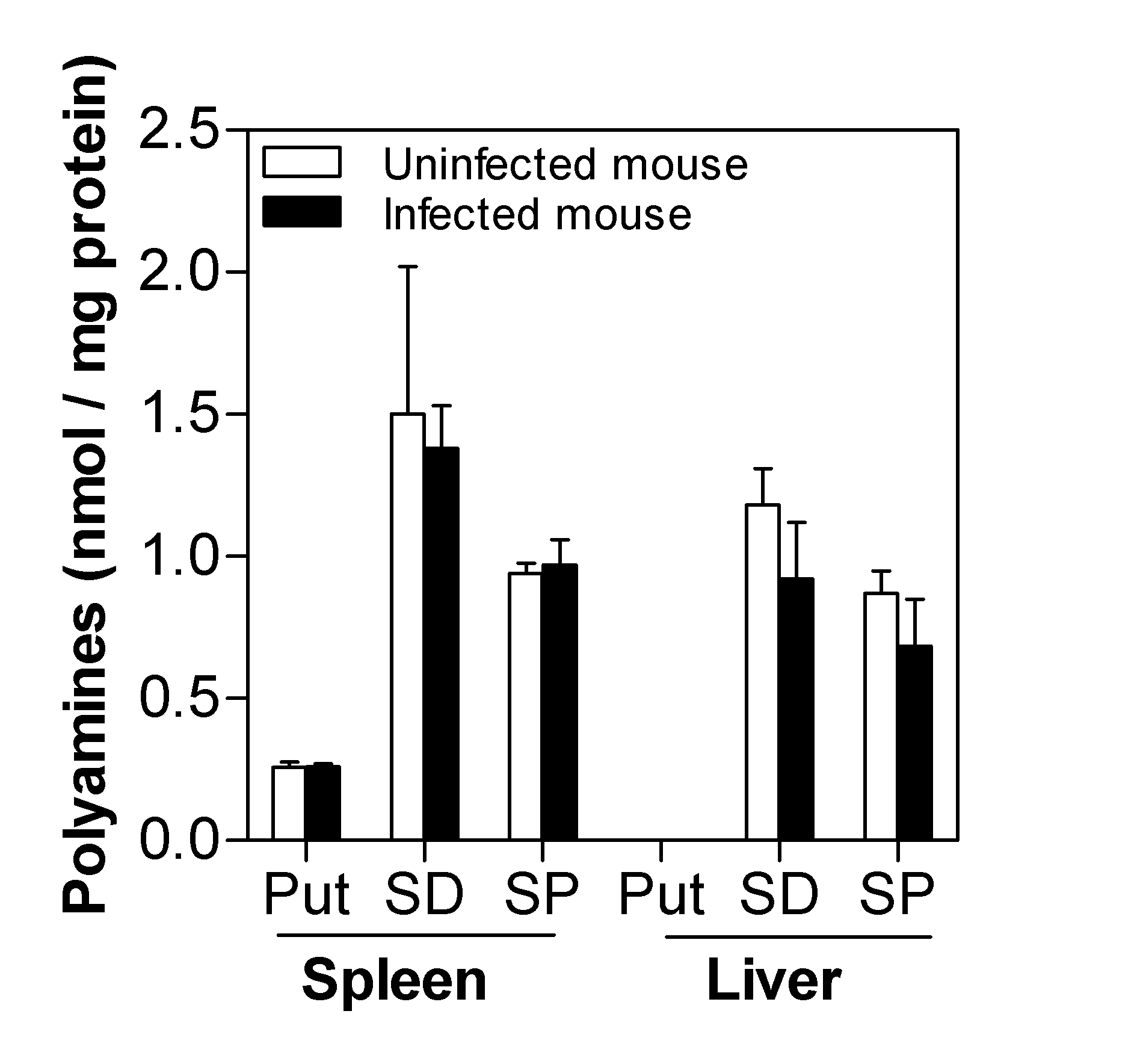

Supplement: Figure S1 — Polyamine content in spleen and liver tissue in L. donovani infected mice. The concentration of polyamines in spleen and liver from groups of 5 uninfected mice (open bars) and 5 infected mice (filled bars) is expressed as the mean and standard deviation (error bars) of nmol polyamine per mg protein. The data shown are from a single experiment that is representative of 2 independent experiments. There were no statistically significant differences between the uninfected and infected tissue samples. (TIF) [file ppat.1002417.s001.tif]

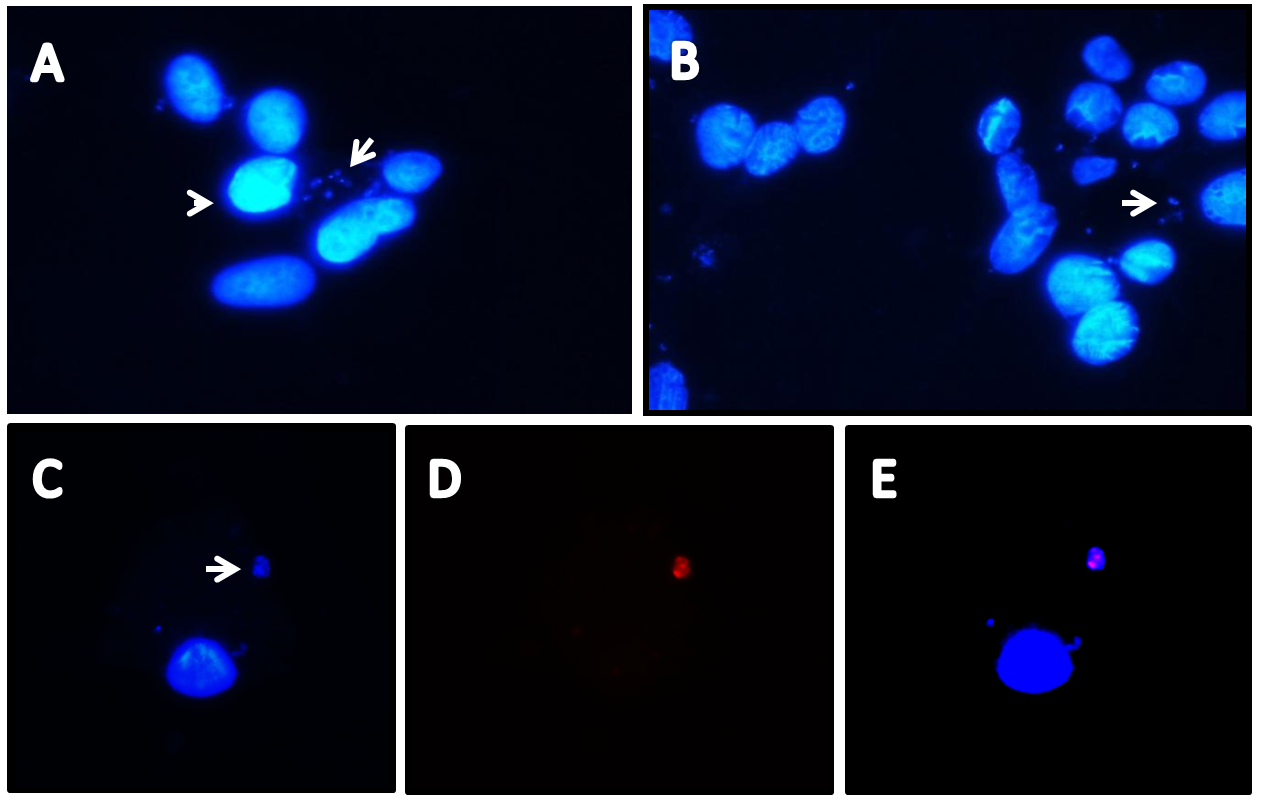

Supplement: Figure S2 — L. donovani infection of BHK cells. BHK cells were infected at 10∶1 ratio with L. donovani promastigotes for 4 hours and then the extracellular parasites were removed by washing 3 times with PBS and once with 0.1% trypsin/EDTA (Gibco) in PBS for 3 min at 37°C. After the last wash the BHK monolayer was detached with 0.25% trypsin and the cells collected by centrifugation at 400 x g for 5 min. The pelleted cells were adjusted to 200,000 cells/200 µL of culture medium, transferred to 4-well chamber slides, and incubated for 24 h at 37°C, 5% CO2. (A-B) Intracellular amastigotes were imaged at 40× magnification after nuclear labeling with 2 µg/mL Hoechst 33342 (Molecular Probes, Invitrogen) for 5 min. A BHK nucleus is shown by an arrowhead and amastigotes are identified by arrows. In some instances the amastigotes are oriented so that the kinetoplast DNA is clearly visible. (C) L. donovani amastigotes in BHK cells imaged at 100X magnification after staining with Hoechst 33342 and (D) 100 nM Lysotracker Red DND-99 (Molecular Probes, Invitrogen) to stain the phagolysosome. (E) Overlay of the Hoechst and Lysotracker Red stained images using the NIS-Elements Software (Nikon) to confirm the intraphagolysosmal location of the amastigotes. (TIF) [file ppat.1002417.s002.tif]

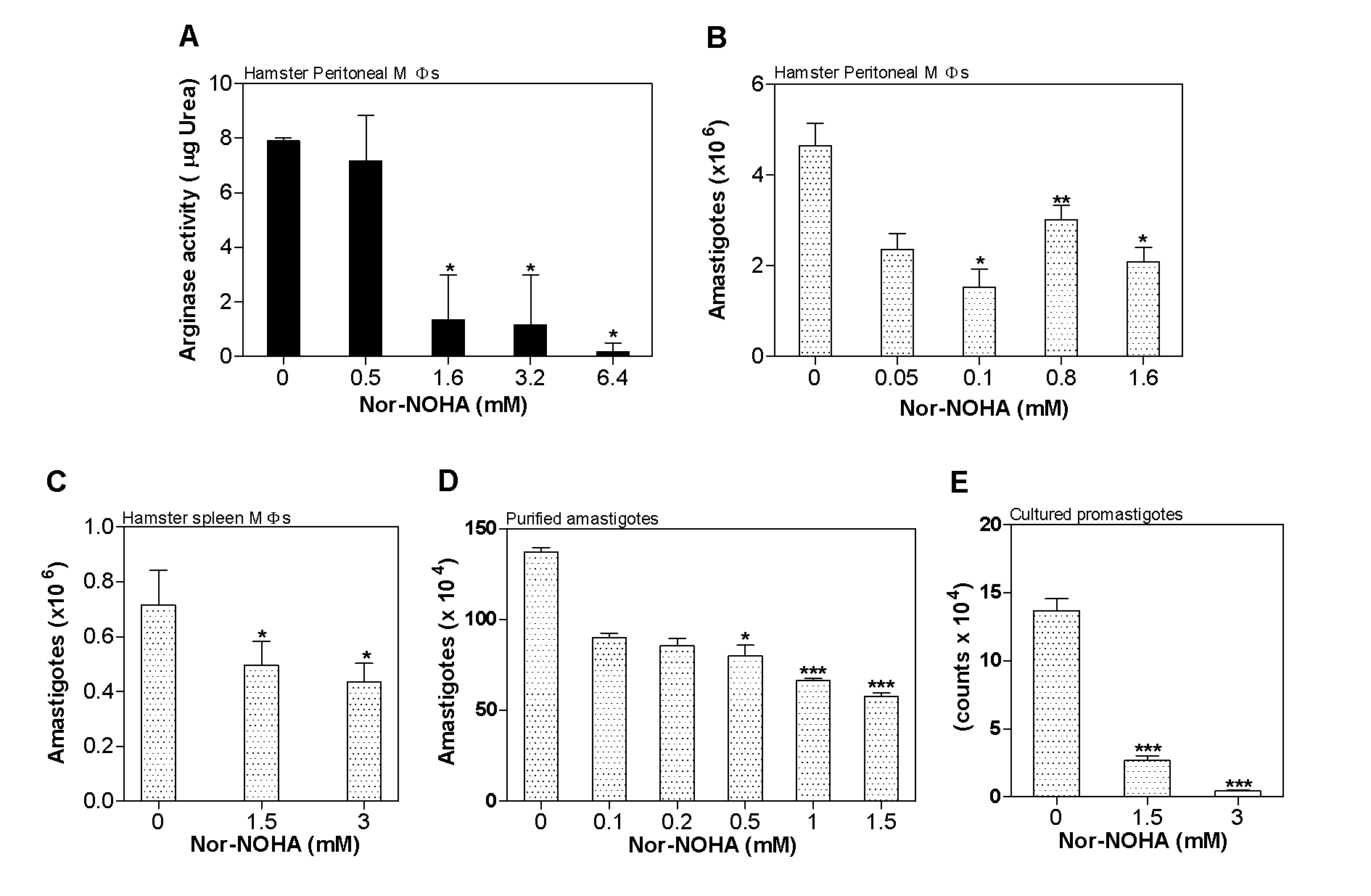

Supplement: Figure S3 — Anti- Leishmania activity of the inhibitor nor-NOHA (Nω-hydroxy-nor-Arginine). (A) Arginase activity was measured in supernatants of L. donovani-infected hamster peritoneal macrophages after incubation with or without nor-NOHA for 48 h. The data is shown as the mean and standard deviation (error bars) of the arginase activity determined by assay of urea production in 100,000 cells. Statistical differences are shown between untreated and treated samples. (B) Number of amastigotes in L. donovani-infected hamster peritoneal macrophages after incubation with or without nor-NOHA for 48 h. The data is shown as the mean and standard deviation (error bars) of the number of parasites determined by luminometry. (C) Number of amastigotes in splenic macrophages isolated from hamsters at 15 days post-infection and untreated or treated ex vivo for 48 h with nor-NOHA. The data is shown as the mean and standard deviation (error bars) of the number of parasites determined by luminometry. (D) Number of amastigotes (purified from infected hamster spleen) after 24 h of in vitro culture with or without nor-NOHA (seeded at 100,000 amastigotes/100 µL). The data is shown as the mean and standard deviation (error bars) of the number of parasites determined by luminometry. (E) Number of promastigotes after 24h of in vitro culture with or without norNOHA (seeded at 100,000 amastigotes/100 µL). The data is shown as the mean and standard deviation (error bars) of the number of parasites determined by luminometry. The data shown for each of the panels is from a single experiment representative of at least 2 independent experiments. The statistical significance of differences between groups in each of the panels is identified by asterisks (*, p<0.05; **, p<0.01; ***, p<0.001). (TIF) [file ppat.1002417.s003.tif]

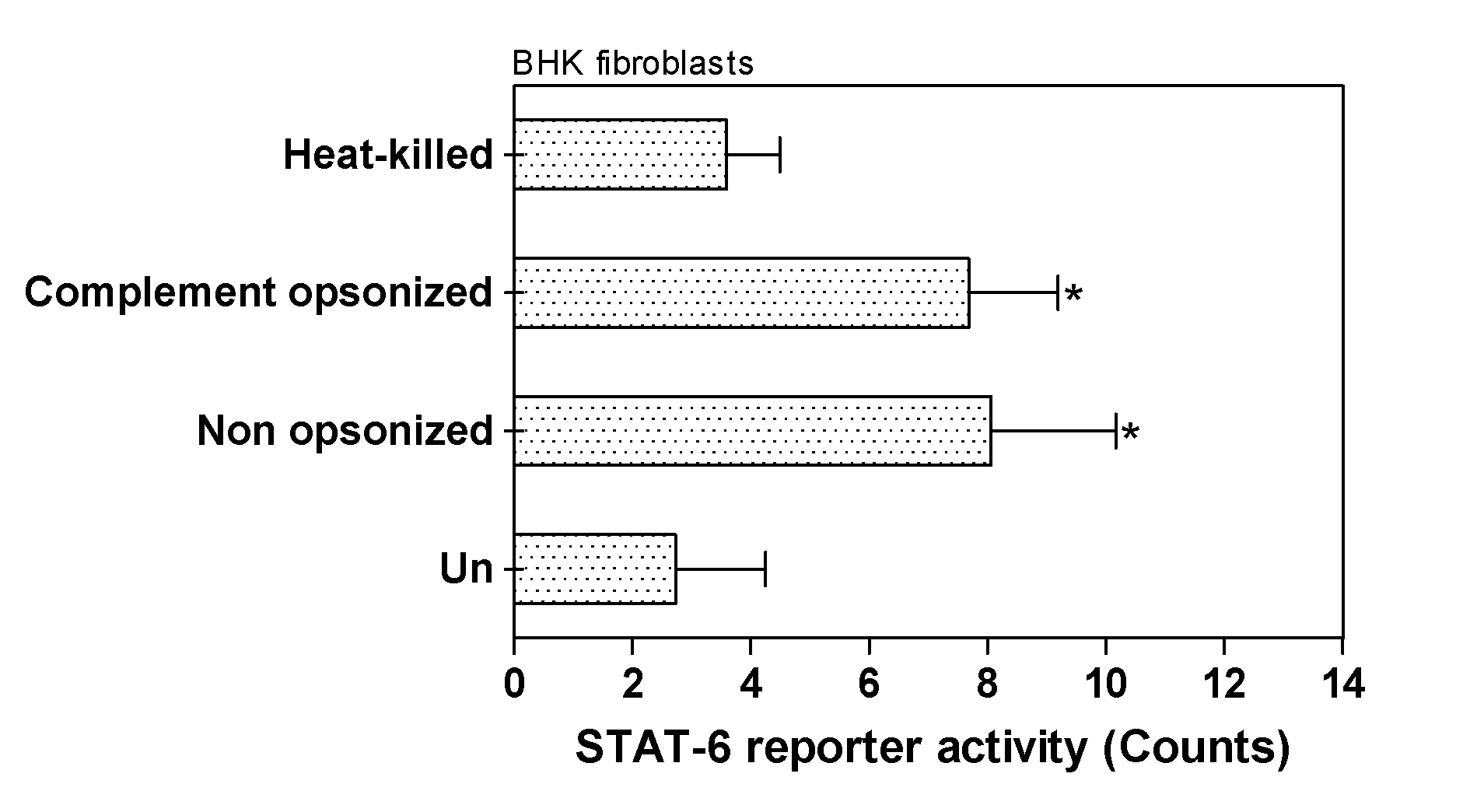

Supplement: Figure S4 — Effect of opsonization on L. donovani -induced STAT-6 activation. BHK cells transfected with a STAT6-luciferase reported vector were exposed or not to unopsonized, or complement opsonized (fresh hamster serum), or heat killed L. donovani promastigotes. Data are presented as the mean and standard deviation (error bars) of the relative light units in uninfected (Un) cells and cells exposed to 10 parasites per cell over 48 hrs of culture. Shown is data from a single experiment that is representative of 2 independent experiments. The statistical significance of differences between uninfected and parasite-exposed groups is identified by asterisks (*, p<0.05). (TIF) [file ppat.1002417.s004.tif]
